# Association of Vitamin C, Thiamine, and Hydrocortisone Infusion With Long-term Cognitive, Psychological, and Functional Outcomes in Sepsis Survivors: A Secondary Analysis of the Vitamin C, Thiamine, and Steroids in Sepsis Randomized Clinical Trial

**DOI:** 10.1001/jamanetworkopen.2023.0380

**Published:** 2023-02-28

**Authors:** Shawniqua Williams Roberson, Samuel Nwosu, Erin M. Collar, Amy Kiehl, Fiona E. Harrison, Julie Bastarache, Jo Ellen Wilson, Matthew F. Mart, Jonathan E. Sevransky, E. Wesley Ely, Christopher J. Lindsell, James C. Jackson

**Affiliations:** 1 Critical Illness, Brain Dysfunction and Survivorship Center, Vanderbilt University Medical Center, Nashville, Tennessee; 2 Department of Neurology, Vanderbilt University Medical Center, Nashville, Tennessee; 3 Department of Biomedical Engineering, Vanderbilt University, Nashville, Tennessee; 4 Department of Biostatistics, Vanderbilt University Medical Center, Nashville, Tennessee; 5 Division of Allergy, Pulmonary and Critical Care Medicine, Vanderbilt University Medical Center, Nashville, Tennessee; 6 Division of Diabetes, Endocrinology and Metabolism, Department of Medicine, Vanderbilt University Medical Center, Nashville, Tennessee; 7 Vanderbilt Brain Institute, Vanderbilt University, Nashville, Tennessee; 8 Department of Psychiatry and Behavioral Sciences, Vanderbilt University Medical Center, Nashville, Tennessee; 9 Division of Pulmonary, Allergy Critical Care and Sleep, Emory University School of Medicine, Atlanta, Georgia; 10 Emory Critical Care Center, Emory Healthcare, Atlanta, Georgia; 11 Veteran’s Affairs Tennessee Valley Geriatric Research Education Clinical Center, Nashville, Tennessee

## Abstract

**Importance:**

Sepsis is associated with long-term cognitive impairment and worse psychological and functional outcomes. Potential mechanisms include intracerebral oxidative stress and inflammation, yet little is known about the effects of early antioxidant and anti-inflammatory therapy on cognitive, psychological, and functional outcomes in sepsis survivors.

**Objective:**

To describe observed differences in long-term cognitive, psychological, and functional outcomes of vitamin C, thiamine, and hydrocortisone between the intervention and control groups in the Vitamin C, Thiamine, and Steroids in Sepsis (VICTAS) randomized clinical trial.

**Design, Setting, and Participants:**

This prespecified secondary analysis reports the 6-month outcomes of the multicenter, double-blind, placebo-controlled VICTAS randomized clinical trial, which was conducted between August 2018 and July 2019. Adult patients with sepsis-induced respiratory and/or cardiovascular dysfunction who survived to discharge or day 30 were recruited from 43 intensive care units in the US. Participants were randomized 1:1 to either the intervention or control group. Cognitive, psychological, and functional outcomes at 6 months after randomization were assessed via telephone through January 2020. Data analyses were conducted between February 2021 and December 2022.

**Interventions:**

The intervention group received intravenous vitamin C (1.5 g), thiamine hydrochloride (100 mg), and hydrocortisone sodium succinate (50 mg) every 6 hours for 96 hours or until death or intensive care unit discharge. The control group received matching placebo.

**Main Outcomes and Measures:**

Cognitive performance, risk of posttraumatic stress disorder and depression, and functional status were assessed using a battery of standardized instruments that were administered during a 1-hour telephone call 6 months after randomization.

**Results:**

After exclusions, withdrawals, and deaths, the final sample included 213 participants (median [IQR] age, 57 [47-67] years; 112 males [52.6%]) who underwent long-term outcomes assessment and had been randomized to either the intervention group (n = 108) or control group (n = 105). The intervention group had lower immediate memory scores (adjusted OR [aOR], 0.49; 95% CI, 0.26-0.89), higher odds of posttraumatic stress disorder (aOR, 3.51; 95% CI, 1.18-10.40), and lower odds of receiving mental health care (aOR, 0.38; 95% CI, 0.16-0.89). No other statistically significant differences in cognitive, psychological, and functional outcomes were found between the 2 groups.

**Conclusions and Relevance:**

In survivors of sepsis, treatment with vitamin C, thiamine, and hydrocortisone did not improve or had worse cognitive, psychological, and functional outcomes at 6 months compared with patients who received placebo. These findings challenge the hypothesis that antioxidant and anti-inflammatory therapy during critical illness mitigates the development of long-term cognitive, psychological, and functional impairment in sepsis survivors.

**Trial Registration:**

ClinicalTrials.gov Identifier: NCT03509350

## Key Points

**Question:**

Does early antioxidant and anti-inflammatory therapy improve the long-term cognitive, psychological, and functional outcomes in adults with sepsis?

**Findings:**

In this secondary analysis of a randomized clinical trial involving 213 survivors of sepsis with respiratory and/or cardiovascular dysfunction, early treatment with vitamin C, thiamine, and hydrocortisone resulted in no improvement in many domains, worse immediate memory scores, and higher odds of posttraumatic stress disorder compared with placebo.

**Meaning:**

Findings of this study suggest that antioxidant and anti-inflammatory therapy does not mitigate the development of long-term cognitive, psychological, and functional impairment in sepsis survivors.

## Teaser

This secondary analysis of a randomized clinical trial reports on the cognitive performance, risk of posttraumatic stress disorder and depression, and functional status 6 months after the administration of a treatment regimen consisting of antioxidants and hydrocortisone infusion in adults with sepsis.

## Introduction

Sepsis is a systemic inflammatory response to infection that frequently leads to multiorgan system failure, increased mortality, and long-term cognitive and functional impairment.^1,2^ Of the almost 2 million sepsis cases in the US annually, nearly 55% require intensive care unit (ICU) admission and 20% to 30% die.^3^ Sepsis survivors are at 3 to 4 times greater risk of moderate to severe cognitive impairment compared with patients who are hospitalized without sepsis.^4^ Sepsis survivors are also at greater risk of postintensive care syndrome (PICS), a collection of symptoms conferring long-term cognitive and psychological deterioration as well as worse quality of life.^4,5,6,7^ While early antibiotic administration, hemodynamic support, fluid resuscitation, and control of the infectious source have been associated with improved mortality,^8,9^ sepsis-associated cognitive, psychological, and functional decline remain a major public health problem.^10,11^

Postsepsis cognitive impairment is postulated to result from a combination of oxidative stress and prolonged neuroinflammation that results from the initial systemic inflammatory response. Increases in reactive oxygen species induce cellular damage, which, when combined with persistent activation of microglia, can contribute to blood-brain barrier dysfunction, loss of synapses, and neuronal cell death.^12,13,14,15^ Vitamin C is a potent antioxidant and may prevent cognitive decline after sepsis by protecting cells against oxidative lipid damage.^16,17^ Humans are among the few mammals that cannot synthesize vitamin C endogenously^18^ and are at risk for vitamin C depletion during sepsis.^19,20,21^ Both vitamin C and hydrocortisone have been associated with decreases in markers of acute inflammation in patients with sepsis^22,23^ and could act synergistically to prevent and repair endothelial cell dysfunction.^24,25,26^ The adverse cognitive and psychiatric effects of vitamin C deficiency in humans have been recognized for centuries.^27^ Thiamine, for its part, is also frequently deficient in sepsis.^28^ Thiamine deficiency increases inflammatory markers^29^ and is associated with cognitive impairment.^30^ These findings suggest that a combination of vitamin C, hydrocortisone, and thiamine may protect patients against postsepsis cognitive impairment. In addition, some studies have suggested a survival advantage from the same therapeutic approach.^28^ However, of 5 large randomized clinical trials,^23,31,32,33,34^ all but 1 failed to demonstrate a survival benefit from vitamin C–based therapy. Although 1 of these trials examined quality of life at 6 months,^34^ no study to date has evaluated the effects of these therapies on postsepsis cognitive and psychological outcomes.

The multicenter, double-blind, placebo-controlled Vitamin C, Thiamine, and Steroids in Sepsis (VICTAS) randomized clinical trial was conducted between August 2018 and July 2019 to test the hypothesis that this combination therapy would improve clinically important outcomes in patients with sepsis-induced respiratory and/or circulatory failure.^35^ The trial was designed a priori to evaluate the primary objective of effects on mortality and ventilator- and vasopressor-free days, which were shown to be unaffected by the treatment regimen.^36^ In addition, the trial was designed for a secondary objective of examining the effects of this combination therapy on the cognitive, psychological, and functional status at the 6-month follow-up of participants in the intervention and control groups, which was the focus of this secondary analysis.^37^ We hypothesized that, among survivors of sepsis requiring vasopressor or ventilator support, early treatment with high-dose vitamin C, thiamine, and hydrocortisone improves cognitive performance, psychological symptoms, and functional status 6 months after randomization.

## Methods

### Study Procedures

Details of the VICTAS trial protocol and analysis have been reported.^35,36,37^ Briefly, adult patients with acute respiratory and/or cardiac dysfunction due to sepsis who required ventilator or vasopressor support were recruited at 43 hospitals across the US. Participants were randomized 1:1 to either the intervention or control group. Participants, investigators, and study team personnel responsible for outcomes assessment were blinded to treatment allocation. The VICTAS trial, including this prespecified secondary analysis, was approved by the institutional review board of the Johns Hopkins Hospital. Participants or their legally authorized representatives provided written informed consent prior to enrollment and randomization. We followed the Consolidated Standards of Reporting Trials (CONSORT) reporting guideline.

The intervention consisted of intravenous vitamin C (1.5 g), thiamine hydrochloride (100 mg), and hydrocortisone sodium succinate (50 mg) administered within 4 hours of randomization and every 6 hours thereafter for up to 96 hours or until death or ICU discharge (whichever occurred first). Participants in the control group received placebo injections of normal saline matched by volume at the same frequency. Participants could be treated with open-label corticosteroids as deemed appropriate by the clinicians; in cases of daily doses greater than or equal to 200 mg of hydrocortisone (or equivalent), investigational hydrocortisone or matching placebo was withheld by the pharmacy. Trained research personnel assessed participants for delirium daily during the intervention period using the Confusion Assessment Method for the ICU in conjunction with the Richmond Agitation-Sedation Scale. All other clinical management of participants, including enteral vitamin supplementation and nutrition, was at the discretion of the clinicians.

### Measurement of Long-term Outcomes

At discharge or at 30 days, whichever occurred first, participants were invited to a follow-up telephone interview at 6 months after randomization. The invitation did not require separate informed consent from the main study but did require an expression of willingness by the participant or participant’s representative to be contacted at approximately 6 months. To facilitate long-term cohort retention, we used a follow-up window of 5 to 8 months. Follow-up assessments were conducted through January 2020.

During the follow-up telephone interview, we used a previously validated^38^ battery of cognitive assessment instruments, including the following: Telephone Confusion Assessment Method^39^ to assess for delirium; Telephone Interview for Cognitive Status^40^ to assess global cognitive function; Wechsler Adult Intelligence Scale, Fourth Edition, Digit Span Subtest to assess attention and working memory capacity; Weschler Memory Scale, Fourth Edition, Logical Memory I and II to examine immediate and delayed memory, respectively; Wechsler Adult Intelligence Scale, Fourth Edition, Similarities Subtest to assess language conceptualization and verbal abstraction; Controlled Oral Word Association Test to assess verbal fluency; and Hayling Sentence Completion Test to assess response inhibition as a form of executive function. Higher scores on these instruments indicate better outcomes. Complete descriptions of these assessments, their respective scoring systems, and score interpretation are provided in eAppendix 1 in Supplement 1.

To assess psychological status, we used the Posttraumatic Stress Disorder 8-item questionnaire (PTSD-8)^41^ and the Patient-Reported Outcomes Measurement Information System Depression 6-item Short Form (PROMIS-6).^42^ Posttraumatic stress disorder (PTSD) screening was anchored to the ICU experience, and participants were considered to have PTSD if they reported a PTSD-8 score of 3 or higher on a 4-point Likert scale in 3 of 4 symptom categories.^41^ Participants were considered to have depression if the total symptom burden on the PROMIS-6 exceeded a T score of 60, which corresponds to moderate depression on commonly used depression measures.^43^ Higher scores on the PTSD-8 and PROMIS-6 indicate worse outcomes. These instruments and their scoring criteria are described in further detail in eAppendix 1 in Supplement 1.

We used the Katz Activities of Daily Living Scale^44^ and the Functional Activities Questionnaire^45^ to assess basic and instrumental activities of daily living, respectively. Higher scores on these instruments indicate worse outcomes. We used the EuroQoL 5-Dimensions 3-Level (EQ-5D-3L)^46^ to assess overall health-related quality of life. A higher score on the EQ-5D-3L reflects better outcomes. We included additional standardized questions to assess health care use since discharge^47^ (eAppendix 1 in Supplement 1).

Telephone interviews lasted approximately 60 minutes. In cases where the participant was unable or unwilling to undergo formal cognitive and psychological assessment by telephone (eg, due to illness, excessive fatigue, or cognitive deterioration), functional status and health care use data were gathered from a designated surrogate. Reasons for inability to participate in individual assessments were recorded.

### Statistical Analysis

Categorical variables were reported using frequencies and proportions. Continuous variables were reported as means with SDs or medians with IQRs. Participants were analyzed according to the group to which they were randomized (ie, intervention vs control). Comparisons between groups, with 2-tailed *P* values, used a multivariable proportional odds logistic regression for ordinal outcomes and a binary logistic regression for dichotomous outcomes. We adjusted for the following covariates: age, sex, race and ethnicity (identified by self-report or researcher observation), educational level, Acute Physiology and Chronic Health Evaluation version II score, presence of diabetes, neurological or cardiovascular comorbidity, number of days requiring mechanical ventilation, and ICU length of stay. *P* < .05 was considered to be statistically significant. For standardized cognitive assessments, scaled scores were used in the statistical analysis.

This secondary analysis reported on the key secondary objective for the VICTAS trial. The trial was explicitly powered for the primary objective and was administratively terminated after recruiting 501 participants. The secondary analysis was specified a priori with the intent to describe the magnitude of differences between groups rather than to focus on hypothesis tests. Therefore, no adjustments for multiplicity were made, and adjusted effect sizes (ie, odds ratios [ORs]) with 95% CIs were reported. Multiple imputation based on predictive mean matching was used to overcome missing covariate data.

Approximately one-third of participants who were interviewed at follow-up were unable or unwilling to complete the full battery of cognitive and psychological assessments (eAppendix 2 and eTable 2 in Supplement 1 describe the completion rates for individual assessments). In less than 7% of included participants who were unable to complete a given assessment due to cognitive impairment, the lowest possible score was assigned. All other missing data were assumed to be missing completely at random.

Sensitivity analysis was performed with a complete case approach. All analyses were conducted between February 2021 and December 2022 using R, version 3.4.3 (R Foundation for Statistical Computing).

## Results

### Enrollment and Patient Characteristics

**Figure 1.**
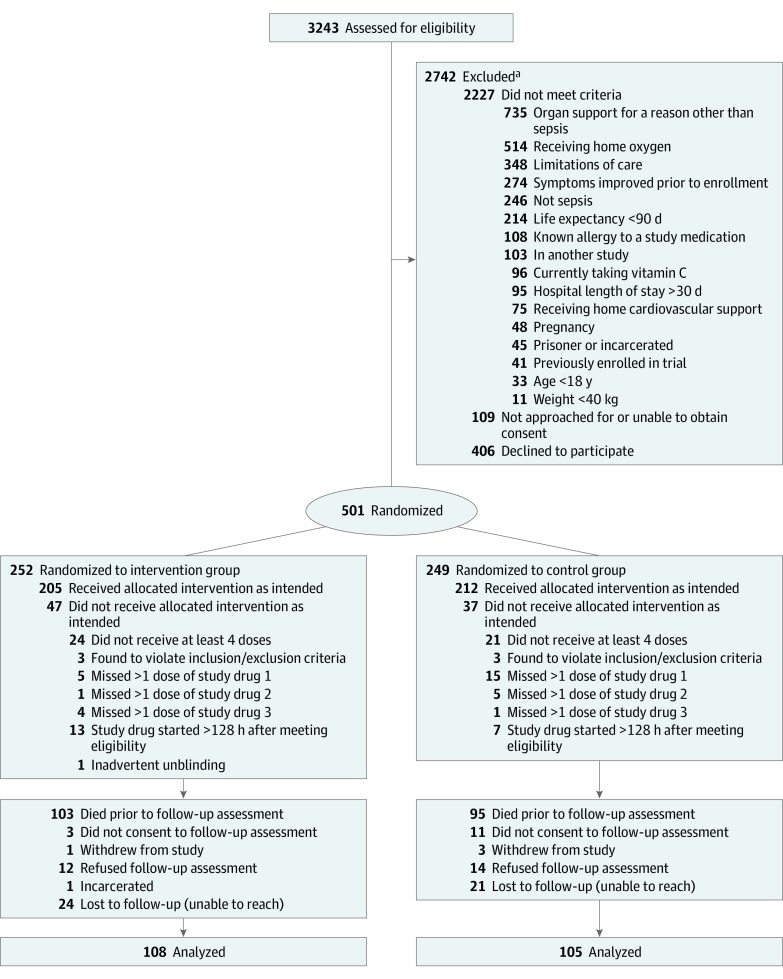
Recruitment, Randomization, and Flow of Participants in the VICTAS Study ^a^Participants could meet multiple criteria; thus, exclusions were not mutually exclusive.

The flowchart of enrollment, randomization, and follow-up of VICTAS trial participants is depicted in Figure 1. Of the 501 participants randomized, 198 (39.5%) died prior to undergoing long-term outcome assessment (196 died within 180 days of randomization and 2 died after 180 days of randomization but before their 5- to 8-month follow-up window ended). Of the remaining 303 participants, 4 (1.3%) withdrew from the study and 40 (13.2%) did not agree to follow-up assessment. Of the 285 participants available for follow-up, 32 (11.2%) could not be reached within their 5- to 8-month follow-up window and 13 (4.6%) had a truncated follow-up window due to administrative termination of the trial. One participant was administratively withdrawn due to incarceration, and 26 (9.1%) refused assessment at follow-up.

The final sample included 213 participants, of whom 108 were randomized to the intervention group and 105 were randomized to the control group. The characteristics of the included participants are described in Table 1 and eTable 1 in Supplement 1, along with participants who died and those for whom outcomes were unavailable (eg, due to withdrawal or loss to follow-up). The included participants had a median (IQR) age of 57 (47-67) years and comprised 101 females (47.4%) and 112 males (52.6%). Data were reported by participant representatives only in 39 cases (18.3%). The intervention and control groups had comparable characteristics (Table 2) except that the intervention group included more White participants than the control group (72 [66.7%] vs 57 [54.3%]). One hundred fifty-seven participants (73.7%) in the follow-up cohort, 72 (68.6%) in the control group, and 85 (78.7%) in the intervention group received parenteral norepinephrine. Sixty-six patients (31.0%) in the follow-up cohort (32 patients [30.5%] from the control group and 34 [31.5%] from the intervention group) received open-label corticosteriods while in the ICU.

**Table 1.** Patient Characteristics Stratified by Follow-up Status

Characteristic	No. (%)			
	Follow-up	No follow-up		Overall
		Died	Ineligible or lost to follow-up^a^	
No. of patients	213	198^b^	90	501
Age, median (IQR), y	57 (47-67)	65 (55-75)	60 (48-70)	62 (50-70)
Sex				
Male	112 (52.6)	117(59.1)	44 (48.9)	273 (54.5)
Female	101 (47.4)	81 (40.9)	46 (51.1)	228 (45.5)
Race and ethnicity^c^				
Black	68 (31.9)	56 (28.3)	26 (28.9)	150 (29.9)
Hispanic or Latino	13 (6.1)	28 (14.1)	15 (16.7)	56 (11.2)
White	129 (60.6)	107 (54.0)	48 (53.3)	284 (56.7)
Other^d^	16 (7.5)	35 (17.7)	16 (17.8)	67 (13.4)
Educational level^e^				
<High school	41 (19.2)	19 (9.6)	11 (12.2)	71 (14.2)
High school diploma or GED	55 (25.8)	28 (14.1)	14 (15.6)	97 (19.4)
Some college	112 (52.6)	36 (18.2)	23 (25.6)	171 (34.1)
Unknown	5 (2.3)	115 (58.1)	42 (46.7)	162 (32.3)
Medical history at enrollment				
BMI, median (IQR)	28 (23-33)	27 (23-33)	27 (23-33)	27 (23-33)
Diabetes	64 (30.0)	61 (30.8)	37 (41.1)	162 (32.3)
Cardiovascular disease	98 (46.0)	117 (59.1)	40 (44.4)	255 (50.9)
Respiratory disease	49 (23.0)	46 (23.2)	16 (17.8)	111 (22.2)
Current cancer	28 (13.1)	54 (27.3)	14 (15.6)	96 (19.2)
Neurological disease	41 (19.2)	38 (19.2)	15 (16.7)	94 (18.8)
APACHE II score, median (IQR)^f^	25 (19-31)	30 (25-35)	24.5 (18-33)	27 (21-33)
SOFA score, median (IQR)^g^	8 (6-10)	10 (8-13)	8 (6-11)	9 (7-12)
Organ support at enrollment				
Vasopressor	93 (43.7)	55 (27.8)	42 (46.7)	190 (37.9)
Ventilator	48 (22.5)	35 (17.7)	20 (22.2)	103 (20.6)
Both	72 (33.8)	108 (54.5)	27 (30.0)	207 (41.3)
Days on mechanical ventilation, median (IQR)	0 (0-3)	1 (0-9)	0 (0-2)	0 (0-4)
ICU admission source				
Emergency	145 (68.1)	132 (66.7)	76 (84.4)	353 (70.5)
Hospital floor	34 (16.0)	45 (22.7)	8 (8.9)	87 (17.4)
Step-down unit	13 (6.1)	3 (1.5)	1 (1.1)	17 (3.4)
Intermediate care	1 (0.5)	3 (1.5)	0	4 (0.8)
Other^h^	20 (9.4)	15 (7.6)	5 (5.6)	40 (8.0)
Length of ICU stay, d	3.00 (2.00-6.00)	5.00 (2.00-12.00)	3.00 (2.00-6.00)	4.00 (2.00-8.00)
Admission reason				
Sepsis	149 (70.0)	137 (69.2)	70 (77.8)	356 (71.1)
Other medical	53 (24.9)	59 (29.8)	18 (20.0)	130 (25.9)
Other surgical	11 (5.2)	2 (1.0)	2 (2.2)	15 (3.0)
Coma- or delirium-free days, median (IQR)	5 (2-5)	3 (1-5)	5(3-5)	4 (2-5)

Abbreviations: APACHE II, Acute Physiology and Chronic Health Evaluation, version 2; BMI, body mass index (calculated as weight in kilograms divided by height in meters squared); GED, General Educational Development; ICU, intensive care unit; SOFA, Sequential Organ Failure Assessment.

^a^ Patients who were ineligible for or lost to follow-up included 4 who withdrew, 26 who declined follow-up assessment, 14 who did not consent to follow-up assessment, 1 who was incarcerated at the time of follow-up contact, and 45 who could not be contacted during their follow-up window despite multiple attempts.

^b^ In a previous report^36^ of primary outcome measures, 196 participants died at 180 days. An additional 2 participants died prior to the end of their 5- to 8-month window for completion of cognitive, psychological, and functional assessments.

^c^ Race and ethnicity were identified by self-report or researcher observation.

^d^ Other race and ethnicity included American Indian or Alaska Native, Asian, Native Hawaiian or other Pacific Islander, mixed race, and other.

^e^ Educational level was reported by patient or surrogate during interview at enrollment and at follow-up. Unknown means the respondent did not know the highest educational level attained by the participant.

^f^ The APACHE II score ranges from 0 to 71, with higher scores indicating greater risk of hospital death. A score of 25 indicates a mortality probability of approximately 50%.

^g^ The SOFA score ranges from 0 to 24, with higher scores indicating greater severity of organ dysfunction. A score between 7 and 9 is associated with a 40% to 50% mortality risk.

^h^ Other included outside hospital, operating suite, inpatient rehabilitation unit, oncology unit, another ICU, emergency department observation unit, nursing home, and urgent care.

**Table 2.** Characteristics of Included Patients Stratified by Group

Characteristic	No. (%)	
	Control group	Intervention group
No. of participants	105	108
Age, median (IQR), y	56 (46-66)	59 (51-68)
Sex		
Male	53 (50.5)	59 (54.6)
Female	52 (49.5)	49 (45.4)
Race and ethnicity^a^		
Black	37 (35.2)	31 (28.7)
Hispanic or Latino	7 (6.7)	6 (5.6)
White	57 (54.3)	72 (66.7)
Other^b^	11 (10.5)	5 (4.6)
Educational level^c^		
<High school	18 (17.1)	23 (21.3)
High school diploma or GED	31 (29.5)	24 (22.2)
Some college	54 (51.4)	58 (53.7)
Unknown	2 (1.9)	3 (2.8)
Medical history at enrollment		
BMI, median (IQR)	29 (23-34)	28 (24-33)
Diabetes	29 (27.6)	35 (32.4)
Cardiovascular disease	46 (43.8)	52 (48.1)
Respiratory disease	23 (21.9)	26 (24.1)
Current cancer	16 (15.2)	12 (11.1)
Neurological disease	22 (21.0)	19 (17.6)
APACHE II score, median (IQR)^d^	25 (16-30)	25 (20-32)
SOFA score^e^	8 (5-10)	8.5 (6-11)
Organ support at enrollment		
Vasopressor	29 (27.6)	19 (17.6)
Ventilator	46 (43.8)	47 (43.5)
Both	30 (28.6)	42 (38.9)
Days on mechanical ventilation, median (IQR)	0 (0-3)	0 (0-3)
ICU admission source		
Emergency	74 (70.5)	71 (65.7)
Hospital floor	15 (14.3)	19 (17.6)
Step-down unit	7 (6.7)	6 (5.6)
Intermediate care	1 (0.5)	0
Other^f^	8 (7.6)	12 (11.1)
Length of ICU stay, median (IQR), d	3 (2-6)	4 (2-6)
Admission reason		
Sepsis	73 (69.5)	76 (70.4)
Other medical	28 (26.7)	25 (23.1)
Other surgical	4 (3.8)	7 (6.5)
Coma- or delirium-free days, median (IQR)	5 (3-5)	4 (2-5)

Abbreviations: APACHE II, Acute Physiology and Chronic Health Evaluation, version 2; BMI, body mass index (calculated as weight in kilograms divided by height in meters squared); GED, General Educational Development; ICU, intensive care unit; SOFA, Sequential Organ Failure Assessment.

^a^ Race and ethnicity were identified by self-report or researcher observation.

^b^ Other race and ethnicity included American Indian or Alaska Native, Asian, Native Hawaiian or other Pacific Islander, mixed race, and other.

^c^ Educational level was reported by patient or surrogate during interview at enrollment and at follow-up. Unknown means the respondent did not know the highest educational level attained by the participant.

^d^ APACHE II score ranges from 0 to 71, with higher scores indicating greater risk of hospital death. A score of 25 indicates a mortality probability of approximately 50%.

^e^ SOFA score ranges from 0 to 24, with higher scores indicating greater severity of organ dysfunction. A score between 7 and 9 is associated with a 40% to 50% mortality risk.

^f^ Other included outside hospital, operating suite, inpatient rehabilitation unit, oncology unit, another ICU, and emergency department observation unit.

### Cognitive Outcomes

**Figure 2.**
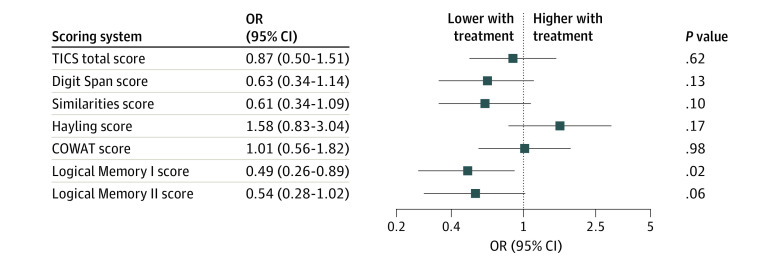
Adjusted Odds Ratios (aORs) for Improvement in 6-Month Cognitive Outcomes With Treatment Participants in the intervention group had worse scores on the Weschler Adult Intelligence Scale IV Logical Memory I subtest (aOR, 0.49; 95% CI, 0.26-0.89), which measures immediate (short-term) memory. Effect sizes for scores on all other cognitive assessments except the Hayling Sentence Completion Test and the Controlled Oral Word Association Test (COWAT) were less than 1, suggesting a tendency toward worse cognitive outcomes with treatment. TICS indicates Telephone Interview for Cognitive Status.

Cognitive outcomes at 6 months are provided in eTable 3 in Supplement 1, and treatment effects are summarized in Figure 2 and eAppendix 3 in Supplement 1. None of the participants who underwent cognitive assessment had delirium at the time of assessment. Treatment with vitamin C, thiamine, and hydrocortisone was generally associated with worse cognitive performance, with statistically significant worsening of immediate memory (Logical Memory I score; adjusted OR [aOR], 0.49; 95% CI, 0.26-0.89; *P* = .02) (Figure 2). The point estimate for the treatment effect was below 1 for all other cognitive assessments (range, 0.49-0.87) except Hayling Sentence Completion Test (aOR, 1.58; 95% CI, 0.83-3.04; *P* = .17) and Controlled Oral Word Association Test (aOR, 1.01; 95% CI, 0.56-1.82; *P* = .98) (Figure 2). Complete case analyses yielded similar effect sizes for all cognitive outcomes (eAppendix 4 and eTable 4 in Supplement 1).

### Psychological and Functional Outcomes

**Figure 3.**
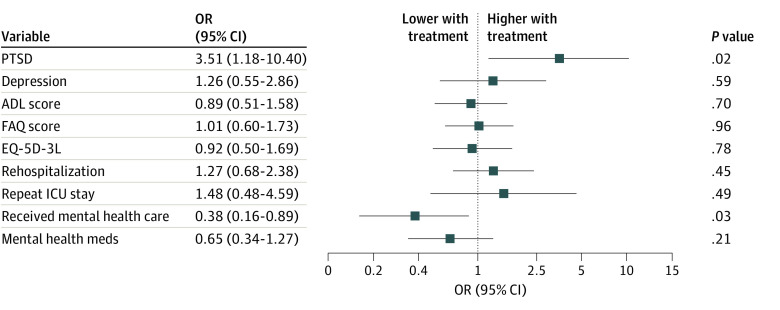
Adjusted Odds Ratios (aORs) for 6-Month Psychological and Functional Outcomes With Treatment Participants in the intervention group had higher odds of having a positive screening result for PTSD (aOR, 3.51; 95% CI, 1.18-10.40) and lower odds of receiving mental health care (aOR, 0.38; 95% CI, 0.16-0.89). Symptom burden (ie, the number of related symptoms reported) and functional capabilities were roughly equivalent between the groups. ADL indicates activities of daily living; EQ-5D-3L, EuroQoL 5-Dimensions 3-Level; FAQ, Functional Activities Questionnaire; ICU, intensive care unit; and PTSD, posttraumatic stress disorder.

The ORs for adverse psychological and functional outcomes are depicted in Figure 3, with counts for each group provided in eTable 3 in Supplement 1 and probabilities plotted in eAppendix 3 in Supplement 1. We detected PTSD in 10 of 105 (9.5%) control participants and 18 of 108 (16.7%) participants in the intervention group (eTable 3 in Supplement 1). Treatment with vitamin C, thiamine, and hydrocortisone was associated with increased odds of PTSD (aOR, 3.51; 95% CI, 1.18-10.40; *P* = .02) (Figure 3). Depression was detected in 50 of 142 (35.2%) participants who completed the PROMIS-6. There was no statistical evidence of a treatment effect on depression (aOR, 1.26; 95% CI, 0.55-2.86; *P* = .59) (Figure 3).

Functional status did not differ between the intervention and control groups. The aOR was 0.89 (95% CI, 0.51-1.58; *P* = .70) for the Katz Activities of Daily Living Scale, 1.01 (95% CI, 0.60-1.73; *P* = .96) for the Functional Activities Questionnaire, and 0.92 (95% CI, 0.50-1.69; *P* = .78) for overall health status on the EQ-5D-3L. The complete case analysis for psychological and functional outcomes yielded similar results (eAppendix 4 and eTable 4 in Supplement 1).

### Health Care Use

The odds of rehospitalization were similar between the intervention and control groups (aOR, 1.09; 95% CI, 0.56-2.12; *P* = .79) (eTable 4 in Supplement 1). Participants in the intervention group had lower odds than those in the control group of receiving formal psychiatric, psychological, or mental health care during the 6 months after discharge (aOR, 0.38; 95% CI, 0.16-0.89; *P* = .03) (Figure 3). The intervention group also had lower odds than the control group of reporting medication use for depression or anxiety, although this difference was not statistically significant (aOR, 0.65; 95% CI, 0.34-1.27; *P* = .21) (Figure 3).

## Discussion

In this prespecified secondary analysis of sepsis survivors in the VICTAS trial, we found no evidence of the benefit of early treatment with vitamin C, thiamine, and hydrocortisone on 6-month cognitive, psychological, and functional outcomes. On the contrary, participants in the intervention group had lower immediate memory scores and higher odds of PTSD compared with participants in the control group. These findings refute the hypothesis that antioxidant and anti-inflammatory therapy during critical illness might mitigate the development of long-term, PICS-related disabilities in survivors of sepsis. The absence of benefit is consistent with results of prior randomized clinical trials that found no improvement in mortality and other hospital-based outcomes with vitamin C therapy.^23,31,32,33,34^ Yet the observation of possible long-term concerns among participants in the intervention group is a signal that warrants caution. Fewer participants in the intervention group than in the control group received mental health care despite these participants more frequently demonstrating possible PTSD. Future studies evaluating the association between acute treatment and longer-term outcomes should consider whether intercurrent therapy is a confounding factor or an outcome in and of itself.

The cognitive scores of both the intervention and control groups were similar in magnitude to those reported in other cohorts of ICU survivors.^47,48^ The median differences in scores for the cognitive assessments were 1 to 2 points on scales ranging from 0 to 10 or 20 (range of 0-100 in Controlled Oral Word Association Test), reflecting a relatively modest potential effect of the intervention on 6-month cognitive outcome. The rates of depression, PTSD, and functional disability were also commensurate with those previously reported^49,50^ and consistent with the outcomes typically observed in PICS.^51,52,53^

Could antioxidant-based therapy truly play a role in increased risk of adverse cognitive, psychological, and functional sequelae among sepsis survivors? The increased odds of PTSD that was observed in the intervention group may have been mediated less by the role of vitamin C as an antioxidant and more by its modulation of neurotransmitter metabolism in the brain. Vitamin C facilitates the conversion of dopamine to norepinephrine (also known as noradrenaline).^54,55^ The latter neurotransmitter is the primary messenger of the locus coeruleus, which mediates physical and emotional responses to stress^56,57^ and facilitates the consolidation of aversive memories.^58,59^ Blockade of norepinephrine receptors enables fear extinction in animals that are subjected to stressful stimuli,^60^ reinforcing the hypothesis that norepinephrine transmission plays an important role in the pathogenesis of PTSD.^61^ If the prevention of vitamin C deficiency during sepsis preserved norepinephrine metabolism, it is possible that this process affected the strength of aversive memory formation during the stressful ICU experience, leading to higher odds of PTSD. Coadministration of hydrocortisone with norepinephrine potentiates fear memory responses^62^ and may have acted synergistically to increase risk of PTSD in the VICTAS trial participants. Alternatively, exogenous norepinephrine and/or corticosteroids may have played a role. A post hoc review of the clinical data revealed that 73.7% of participants in the follow-up cohort (68.6% in the control group, and 78.7% in the intervention group) received parenteral norepinephrine; 31.0% received a concomitant open-label corticosteroid. The differential roles of exogenous vs endogenous neurotransmitters in PTSD pathogenesis have yet to be elucidated. The lower rate of mental health care and higher rate of rehospitalization in the intervention group may also have influenced the risk of PTSD. Regardless of whether the difference in outcomes between groups is directly associated with treatment, the observed rate of PTSD (13.1% [28 of 213 participants in the follow-up cohort) was higher than the past-year prevalence of 3.5% to 6.1% in the general population,^63,64^ lending further support to the Society of Critical Care Medicine recommendations for post-ICU screening of PTSD and other PICS-related symptoms.^65^

Putative mechanisms of postsepsis cognitive decline include intracerebral oxidative stress, inflammation, and endothelial dysfunction.^66^ Vitamin C, hydrocortisone, and thiamine protect neurons from oxidative damage and stem the systemic inflammatory response and thus were expected to decrease the burden of long-term neurological compromise.^16^ Yet we found no clinical evidence of such an effect in this trial. It is possible that the dose, timing, or duration of therapy was insufficient to provide adequate cellular protection. The intervention was limited to the ICU stay (median of 3 days among the follow-up cohort). Mouse models of sepsis suggest that, although vitamin C depletion occurs during the acute illness period,^19^ there are distinct phases of the neuroinflammatory response after injury.^67^ Long-term effects are observed in the central nervous system after continuous activation of microglial cells.^68,69^ Individuals may depend on prolonged vitamin C repletion throughout the recovery period to counteract the oxidative burden and protect against postinjury cognitive decline. Future studies with long-term vitamin C supplementation after sepsis may test this hypothesis.

Sedation and paralysis practices—in particular, number of ICU days with opiates or neuromuscular blockade—may contribute to psychiatric symptoms after critical illness.^70^ Benzodiazepines are factors in increased risk of ICU delirium,^71^ which is also associated with long-term cognitive impairment.^47^ Daily sedation interruption and similar modern critical care bundles and practices limit this risk.^72^ Future studies evaluating the effect of acute therapeutic measures on long-term cognitive and psychiatric outcomes should track adherence to Society of Critical Care Medicine guidelines on the management of pain, agitation, and delirium.^73^

### Limitations

This study has several limitations. First, interpretation was restricted to those who survived sepsis. How survivorship influenced the interpretation of results was unclear, although understanding the differences in patient-reported outcomes based on survivorship remains valuable. Second, a common problem among studies of cognitive and psychological outcomes after critical illness is the unplanned nature of inclusion and the resultant difficulty in adequately assessing for premorbid cognitive and psychological disorders. Despite randomization, there could be baseline differences in cognitive measures among participants in the follow-up cohort that cannot be quantified or controlled in the analysis. We did not collect baseline cognitive or psychological data, which warrants the cautious interpretation of the results.

Third, 40 of 303 eligible participants (13.2%) did not consent to follow-up assessment. The fact that trial participants were not automatically included in this secondary analysis and had to express willingness to participate may have increased attrition. Furthermore, the VICTAS trial was administratively terminated due to withdrawal of funding, leading to a truncated follow-up window for a small proportion (4.6%) of eligible participants. Nonetheless, we were able to obtain at least partial follow-up assessments for approximately 85% of eligible participants, and these sepsis survivors did not differ in characteristics from those who withdrew, refused assessment, or were ultimately lost to follow-up. Some participants were unable or unwilling to complete a few of the assessments, and the rates of missing data reached nearly 25% for several assessments. Among participants who were included in the follow-up cohort, the most common reason for failure to complete an assessment was patient refusal (eTable 2 in Supplement 1). The rates of missing data were similar between the intervention and control groups, and a separate analysis using a complete case approach yielded similar findings. These observations suggest that completion rates did not significantly affect the overall comparisons. Fourth, for pragmatic purposes, we selected brief screening tools as psychological outcome measures. Although these tools are well accepted and have high sensitivity and specificity for detection of PTSD and depression, formal diagnosis requires in-depth evaluation by a trained mental health professional.

## Conclusions

In the VICTAS trial, for survivors of sepsis who were treated early with vitamin C, thiamine, and hydrocortisone in the ICU, the cognitive, psychological, and functional outcomes at the 6-month follow-up either were unaffected by the regimen or were worse than for patients who received placebo. These results do not support antioxidant and anti-inflammatory therapy and suggest that antioxidant and anti-inflammatory therapy does not mitigate the development of long-term cognitive, psychological, and functional impairment in patients with sepsis who require cardiovascular or respiratory support in the ICU.

## Notes

Supplement 1. **eAppendix 1.** Descriptions and Scoring Methods of Outcome Assessment Tools **eTable 1.** Demographic and Clinical Characteristics of the Full Cohort of Included Participants, Stratified by Treatment Group **eAppendix 2.** Missingness and Reasons for Assessment Non-Completion **eTable 2.** Reasons for Assessment Non-Completion **eTable 3.** Outcomes at 6 Months in Sepsis Survivors With and Without Vitamin C, Thiamine & Hydrocortisone Therapy **eAppendix 3.** Evaluation of the Proportional Odds Assumption and Probability Plots **eAppendix 4.** Adjusted Models (Complete Case Analysis) **eTable 4.** Effect of Vitamin C, Thiamine & Hydrocortisone on 6-Month Outcomes After Sepsis
eReferences

Supplement 2.
Nonauthor Collaborators

Supplement 3.
Data Sharing Statement
