# Error in the Byline

**DOI:** 10.1001/jamanetworkopen.2023.12173

**Published:** 2023-04-20

In the Original Investigation titled “Association of Vitamin C, Thiamine, and Hydrocortisone Infusion With Long-term Cognitive, Psychological, and Functional Outcomes in Sepsis Survivors: A Secondary Analysis of the Vitamin C, Thiamine, and Steroids in Sepsis Randomized Clinical Trial,” published February 28, 2023, Ms Kiehl’s name should be listed as Amy L. Kiehl, MA. This article has been corrected.^1^
