# Supplementary material for: Association of Vitamin C, Thiamine, and Hydrocortisone Infusion With Long-term Cognitive, Psychological, and Functional Outcomes in Sepsis Survivors: A Secondary Analysis of the Vitamin C, Thiamine, and Steroids in Sepsis Randomized Clinical Trial
Source: JAMA Netw Open. 2023 Feb 28;6(2):e230380. doi: 10.1001/jamanetworkopen.2023.0380 (PMC9975932; doi:10.1001/jamanetworkopen.2023.0380)
Supplement: Supplement 1. — eAppendix 1. Descriptions and Scoring Methods of Outcome Assessment Tools eTable 1. Demographic and Clinical Characteristics of the Full Cohort of Included Participants, Stratified by Treatment Group eAppendix 2. Missingness and Reasons for Assessment Non-Completion eTable 2. Reasons for Assessment Non-Completion eTable 3. Outcomes at 6 Months in Sepsis Survivors With and Without Vitamin C, Thiamine & Hydrocortisone Therapy eAppendix 3. Evaluation of the Proportional Odds Assumption and Probability Plots eAppendix 4. Adjusted Models (Complete Case Analysis) eTable 4. Effect of Vitamin C, Thiamine & Hydrocortisone on 6-Month Outcomes After Sepsis eReferences [file jamanetwopen-e230380-s001.pdf]

Supplemental Online Content

Williams Roberson S, Nwosu S, Collar EM, et al; VICTAS Investigators. Association of vitamin C, thiamine, and hydrocortisone infusion with long-term cognitive, psychological, and functional outcomes in sepsis survivors: a secondary analysis of the Vitamin C, Thiamine, and Steroids in Sepsis randomized clinical trial. *JAMA Netw Open*. 2023;6(2):e230380. doi:10.1001/jamanetworkopen.2023.0380

eAppendix 1. Descriptions and Scoring Methods of Outcome Assessment Tools

eTable 1. Demographic and Clinical Characteristics of the Full Cohort of Included Participants, Stratified by Treatment Group

eAppendix 2. Missingness and Reasons for Assessment Non-Completion

eTable 2. Reasons for Assessment Non-Completion

eTable 3. Outcomes at 6 Months in Sepsis Survivors With and Without Vitamin C, Thiamine & Hydrocortisone Therapy

eAppendix 3. Evaluation of the Proportional Odds Assumption and Probability Plots

eAppendix 4. Adjusted Models (Complete Case Analysis)

eTable 4. Effect of Vitamin C, Thiamine & Hydrocortisone on 6-Month Outcomes After Sepsis

eReferences

This supplementary material has been provided by the authors to give readers additional information about their work.

© 2023 Williams Roberson S et al. *JAMA Network Open.*

eAppendix 1: Descriptions and Scoring Methods of Outcome Assessment Tools

Measurement of Long-Term Outcomes

Prior to administering neuropsychological assessments in VICTAS, all evaluators were trained in strict and consistent administration of the protocol. After completing training and undergoing an observation period, these evaluators were only then able to perform assessments which were carefully scrutinized. Study staff included 8 individuals, all who had previous extensive experience in the administration of neuropsychological testing via telephone. The neuropsychological tests were performed in a standardized format across all participants in intervention and control groups, and study team members responsible for the assessments were blinded to treatment allocation.

When the participant was unable or unwilling to undergo formal cognitive and psychological assessment by telephone (e.g., due to illness, excessive fatigue, or cognitive deterioration), functional status and healthcare utilization data were gathered from a designated surrogate. We only collected information from surrogates that they would have the capacity to provide based on behavioral observation, e.g. activities of daily living, rehospitalization rates etc. We used the same instruments with language modification to elicit a third-party response. We collected quality of life data directly from individual participants.

Cognitive Assessment Tools

The **Telephone Confusion Assessment Method (T-CAM)**[1] is a 4-point assessment to evaluate presence or absence of delirium during the telephone interview based on:

1. fluctuations in mental status
2. ability to focus attention during the telephone interview
3. level of consciousness and
4. organization/coherence of thought

Delirium is considered present if items 1 AND 2 AND either 3 or 4 are abnormal.

The **Telephone Interview for Cognitive Status (TICS)**[2] is an 11-item screen to assess orientation, short term memory, calculations, language and attention. Scores range from 0 to 41, with higher scores reflecting better performance.

The **WAIS-IV Digit Span Subtest**[3] is a subtest of the Weschler Adult Intelligence Scale that can be used to assess attention and working memory capacity. Participants are given several series of numbers in increasing lengths and instructed to repeat the numbers forward (part 1), backward (part 2), or in a sequence from lowest to highest (part 3). Scores are based on number of correct responses and scaled based on age to range from 0 to 19. Higher scores reflect better performance.

The **Weschler Memory Scale IV Logical Memory I & II Subtests**[4] examine immediate and delayed declarative memory, respectively. The examiner reads a brief story and instructs the participant to repeat the story immediately and again later during the assessment interview. We used two stories, tailored based on the participants' ages. Scores are based on number of salient items included within each story and range from 0 to 19 after combining stories and scaling for age. Higher scores reflect better performance.

The **WAIS-IV Similarities Subtest**[3] is an 18-question survey used to assess language conceptualization and verbal abstraction. Participants are given pairs of words and asked to explain how they are similar (e.g. a HORSE and a TIGER are both animals). Up to two points can be given for each response, for a total score ranging from 0 to 36. Higher scores reflect better performance.

The **Controlled Oral Word Association (COWA) Test**[5] consists of three trials wherein the participant is given a letter prompt (F, A, and S) and requested to list aloud as many words as possible beginning with the specified letter within a 60 second period. This test assesses verbal fluency. One point is given for each unique word (excluding proper nouns), and the scores for the three trials are summed. This total sum is used to find a scaled score and a t-score. Higher scores reflect better performance.

The **Hayling Sentence Completion Test**[6] assesses response inhibition as a form of executive function. Participants listen to a series of sentences with the last word missing and are instructed to complete the sentence with a word that is congruent with the meaning of the sentence. After 15 trials, the participant is then instructed to complete the next 15 sentences with words

© 2023 Williams Roberson S et al. *JAMA Network Open.*

that are *incongruent* with the sentence prompts. For example, in response to the prompt "London is a very busy…" participants might respond with "banana" instead of "city" or "town." Scores are based on response time, number and types of errors, and are scaled on a range from 0 to 10 with higher scores reflecting better performance.

Psychological Assessment Tools

The **PTSD-8**[7] was derived from the first half of the Harvard Trauma Questionnaire Part IV,[8] which corresponds to the DSM-IV criteria for post-traumatic stress disorder. There are four items evaluating intrusion symptoms, two items evaluating avoidance symptoms and two items evaluating hypervigilance symptoms. Item responses are rated on a four-point Likert scale ('not at all' (1), 'a little' (2), 'quite a bit' (3), and 'all the time' (4)). A score of 3 or greater on any item within each of the 3 categories, in relation to a serious or life-threatening event causing intense fear, helplessness or horror that cause clinically significant distress or impairment is consistent with a diagnosis of PTSD. The summed score across all 8 items provides an indication of symptom severity, with higher numbers indicating worse outcome. In the VICTAS study, participants were asked to evaluate the frequency of symptoms over the preceding 6 months, specifically in relationship to their ICU experience.

The **Patient-Reported Outcome Measurement Information System (PROMIS) Depression** item banks were developed as part of the NIH Roadmap for Medical Research Initiative.[9] The PROMIS Depression item banks assess self-reported negative mood, views of self and social cognition, as well as decreased positive affect and engagement. Somatic symptoms are not included, which eliminates consideration of these items' confounding effects when assessing patients with comorbid physical conditions. The depression short forms are universal rather than disease-specific. All assess depression over the past seven days.

The six items presented on the PROMIS Depression 6a Short Form are rated on a scale of 1 (Never) to 5 (Always) and the total sum of scores is converted to a T-score for each participant. The T-score rescales the raw total into a standardized score with a mean of 50 and a standard deviation of 10. Higher scores reflect worse symptoms. A T-score of 60 corresponds to 1 standard deviation (SD) about the population mean and, based on crosswalk with analogous depression screening instruments,[10] is consistent with the presence of moderate depression.

Functional Assessment Tools

The Katz **Activities of Daily Living Score**[11] (ADLS) is a brief inventory designed to assess an individual's current ability to perform routine activities without assistance. Activities addressed include eating, bathing, dressing, toileting, transferring, walking and continence. Activities are rated on a scale of 0 (no dependence) to 2 (total dependence). Higher scores reflect greater level of impairment.

The **Functional Activities Questionnaire (FAQ)**[12] is a 10-question survey designed to assess level of impairment in instrumental activities of daily living such as buying groceries and managing finances. Items are scored on a scale of 0 (Normal function) to 3 (Completely Dependent). and a total score is derived from the sum of all individual item scores. Higher total scores reflect worse impairment.

The **EuroQol-5D**[13] is designed to measure health-related quality of life. It can be used in a wide variety of health conditions. It is divided into two parts: 1) descriptive ratings of level of difficulty in 5 domains (mobility, self-care, usual activities, pain/discomfort and anxiety/depression) and 2) a visual analog scale (VAS) of overall self-perception of health. Items in part 1 are rated on a scale of 1 (no problems) to 3 (very severe problems), and the VAS provides a single overall rating from 0 (worst imaginable health) to 100 (best imaginable health). For the purposes of the current analysis, we report the responses on part 2. Higher scores represent better perception of an individual's own health.

In our **Healthcare Utilization Survey** we asked additional questions about rehospitalization, repeat ICU stays, receipt of mental health care since discharge from the sepsis admission. We also collected information about current use of medications for depression or anxiety.

© 2023 Williams Roberson S et al. *JAMA Network Open.*

eTable 1: Demographic and Clinical Characteristics of the Full Cohort of Included Participants, Stratified by Treatment Group

	**Follow-Up Cohort** *No. (%)*		**Died** *No. (%)*		**Ineligible or Lost to Follow-Up** *No. (%)*		**Overall** *No. (%)*	
	**Control (N=105)**	**Treatment (N=108)**	**Control (N=95)**	**Treatment (N=103)**	**Control (N=49)**	**Treatment (N=41)**	**Control (N=249)**	**Treatment (N=252)**
**Age**, median [Q1, Q3], years	56.0 [46.0, 66.0]	59.0 [51.0, 68.3]	67.0 [57.0, 77.0]	63.0 [54.0, 71.0]	62.0 [50.0, 72.0]	56.0 [43.0, 69.0]	61.0 [50.0, 72.0]	62.0 [50.8, 69.3]
**Sex**								
Male	53 (50.5%)	59 (54.6%)	59 (62.1%)	58 (56.3%)	22 (44.9%)	22 (53.7%)	134 (53.8%)	139 (55.2%)
Female	52 (49.5%)	49 (45.4%)	36 (37.9%)	45 (43.7%)	27 (55.1%)	19 (46.3%)	115 (46.2%)	113 (44.8%)
**Race**								
Caucasian	57 (54.3%)	72 (66.7%)	55 (57.9%)	52 (50.5%)	23 (46.9%)	25 (61.0%)	135 (54.2%)	149 (59.1%)
Black	37 (35.2%)	31 (28.7%)	27 (28.4%)	29 (28.2%)	16 (32.7%)	10 (24.4%)	80 (32.1%)	70 (27.8%)
Other	11 (10.5%)	5 (4.6%)	13 (13.7%)	22 (21.4%)	10 (20.4%)	6 (14.6%)	34 (13.7%)	33 (13.1%)
**Ethnicity**								
Hispanic or Latino	7 (6.7%)	6 (5.6%)	8 (8.4%)	20 (19.4%)	9 (18.4%)	6 (14.6%)	24 (9.6%)	32 (12.7%)
**Education**								
Less than High School	18 (17.1%)	23 (21.3%)	10 (10.5%)	9 (8.7%)	7 (14.3%)	4 (9.8%)	35 (14.1%)	36 (14.3%)
High School or GED	31 (29.5%)	24 (22.2%)	16 (16.8%)	12 (11.7%)	8 (16.3%)	6 (14.6%)	55 (22.1%)	42 (16.7%)
Some College	54 (51.4%)	58 (53.7%)	16 (16.8%)	20 (19.4%)	12 (24.5%)	11 (26.8%)	82 (32.9%)	89 (35.3%)
Unknown[a]	2 (1.9%)	3 (2.8%)	53 (55.8%)	62 (60.2%)	22 (44.9%)	20 (48.8%)	77 (30.9%)	85 (33.7%)
**Medical history at enrollment**								
BMI, Median [Q1, Q3]	28.5 [22.6, 34.1]	27.6 [24.2, 33.0]	25.5 [22.5, 32.6]	27.1 [23.6, 32.9]	27.9 [23.5, 34.5]	27.2 [24.1, 32.1]	26.8 [22.7, 33.4]	27.3 [23.9, 32.8]
Diabetes	29 (27.6%)	35 (32.4%)	28 (29.5%)	33 (32.0%)	20 (40.8%)	17 (41.5%)	77 (30.9%)	85 (33.7%)
Cardiovascular disease	46 (43.8%)	52 (48.1%)	58 (61.1%)	59 (57.3%)	22 (44.9%)	18 (43.9%)	126 (50.6%)	129 (51.2%)
Respiratory disease	23 (21.9%)	26 (24.1%)	25 (26.3%)	21 (20.4%)	8 (16.3%)	8 (19.5%)	56 (22.5%)	55 (21.8%)
Current cancer	16 (15.2%)	12 (11.1%)	31 (32.6%)	23 (22.3%)	8 (16.3%)	6 (14.6%)	55 (22.1%)	41 (16.3%)
Neurological disease	22 (21.0%)	19 (17.6%)	16 (16.8%)	22 (21.4%)	10 (20.4%)	5 (12.2%)	48 (19.3%)	46 (18.3%)
**APACHE II Score**, median [Q1, Q3]	25.0 [16.0, 30.0]	25.0 [20.0, 32.0]	30.0 [24.0, 36.0]	30.0 [26.0, 35.0]	24.5 [17.8, 33.5]	24.5 [18.8, 31.5]	27.0 [19.0, 33.0]	27.0 [22.0, 33.0]
**SOFA Score**, median [Q1, Q3]	8.00 [5.00, 10.0]	8.50 [6.00, 11.0]	10.0 [8.00, 12.5]	10.0 [8.00, 13.0]	8.00 [6.00, 11.0]	8.00 [5.00, 10.0]	9.00 [6.00, 11.0]	9.00 [7.00, 12.0]
**Organ support at enrollment**								
Ventilator	29 (27.6%)	19 (17.6%)	15 (15.8%)	20 (19.4%)	10 (20.4%)	10 (24.4%)	54 (21.7%)	49 (19.4%)
Vasopressor	46 (43.8%)	47 (43.5%)	27 (28.4%)	28 (27.2%)	24 (49.0%)	18 (43.9%)	97 (39.0%)	93 (36.9%)
Both	30 (28.6%)	42 (38.9%)	53 (55.8%)	55 (53.4%)	14 (28.6%)	13 (31.7%)	97 (39.0%)	110 (43.7%)
**Days on mechanical ventilation,** median [Q1, Q3]	0 [0, 3.00]	0 [0, 3.00]	1.00 [0, 5.50]	1.00 [0, 11.0]	0 [0, 2.00]	0 [0, 2.00]	0 [0, 3.00]	0 [0, 5.00]
**ICU Admission Source**								
Emergency	74 (70.5%)	71 (65.7%)	67 (70.5%)	65 (63.1%)	43 (87.8%)	33 (80.5%)	184 (73.9%)	169 (67.1%)
Hospital Floor	15 (14.3%)	19 (17.6%)	22 (23.2%)	23 (22.3%)	3 (6.1%)	5 (12.2%)	40 (16.1%)	47 (18.7%)
Step Down Unit	7 (6.7%)	6 (5.6%)	1 (1.1%)	2 (1.9%)	0 (0%)	1 (2.4%)	8 (3.2%)	9 (3.6%)
Intermediate Care	1 (1.0%)	0 (0%)	0 (0%)	3 (2.9%)	0 (0%)	0 (0%)	1 (0.4%)	3 (1.2%)
Other	8 (7.6%)	12 (11.1%)	5 (5.3%)	10 (9.7%)	3 (6.1%)	2 (4.9%)	16 (6.4%)	24 (9.5%)
**Length of ICU Stay,**	3.00 [2.00, 6.25]	4.00 [2.00, 6.00]	5.00 [2.00, 12.0]	5.00 [2.00, 12.0]	3.00 [2.00, 6.00]	3.00 [2.00, 6.00]	4.00 [2.00, 8.00]	4.00 [2.00, 8.00]

© 2023 Williams Roberson S et al. *JAMA Network Open.*

	Follow-Up Cohort *No. (%)*		Died *No. (%)*		Ineligible or Lost to Follow-Up *No. (%)*		Overall *No. (%)*	
	**Control (N=105)**	**Treatment (N=108)**	**Control (N=95)**	**Treatment (N=103)**	**Control (N=49)**	**Treatment (N=41)**	**Control (N=249)**	**Treatment (N=252)**
median [Q1, Q3]								
**Admission Reason**								
Sepsis	73 (69.5%)	76 (70.4%)	62 (65.3%)	75 (72.8%)	41 (83.7%)	29 (70.7%)	176 (70.7%)	180 (71.4%)
Other Medical	28 (26.7%)	25 (23.1%)	31 (32.6%)	28 (27.2%)	8 (16.3%)	10 (24.4%)	67 (26.9%)	63 (25.0%)
Other Surgical	4 (3.8%)	7 (6.5%)	2 (2.1%)	0 (0%)	0 (0%)	2 (4.9%)	6 (2.4%)	9 (3.6%)
**Coma/Delirium Free Days**, median [Q1, Q3]	5.00 [3.00, 5.00]	4.00 [2.00, 5.00]	2.00 [1.00, 5.00]	3.00 [1.00, 5.00]	4.00 [3.00, 5.00]	5.00 [2.75, 5.00]	4.00 [2.00, 5.00]	4.00 [2.00, 5.00]

a. Premorbid education level was assessed at enrollment and was not known by surrogates in 53% of cases. This information was requested directly from survivors at 6-month follow-up. Participants who died, withdrew, declined or were lost to follow-up did not have the second education assessment.

© 2023 Williams Roberson S et al. *JAMA Network Open.*

eAppendix 2: Missingness and Reasons for Assessment Non-Completion

We report in the following tables the number of included participants not completing each cognitive assessment and reasons for non-completion. Participants who were unable to complete an assessment due to cognitive incapability were assigned the lowest possible score. Scores for the remainder of incomplete assessments were imputed using a predictive mean matching algorithm, as follows: Using the 'fit.mult.impute' function from the 'Himisc' package in R, we predicted the missing covariate values based on the real observed data (i.e. non-missing data), finding matches for the missing values. The regression models were fit to the observed data, then the missing values for each covariate were matched to the closest set of observed values based on the regression fit, which we then imputed based on those closest set of observed values. We repeated this imputation 10 times, where the imputed values were averaged over the 10 number of imputations. Note, only covariate values were imputed; outcome variables were not imputed.

© 2023 Williams Roberson S et al. *JAMA Network Open.*

eTable 2: Reasons for Assessment Non-Completion

	Control (N=105)	Treatment (N=108)	Overall (N=213)
**TICS**			
Patient Died[a]	0 (0%)	1 (0.9%)	1 (0.5%)
Patient Withdrew	1 (1.0%)	1 (0.9%)	2 (0.9%)
Suspected drug/alcohol influence	0 (0%)	0 (0%)	0 (0%)
Patient cognitively incapable	5 (4.8%)	8 (7.4%)	13 (6.1%)
Patient emotionally incapable	1 (1.0%)	1 (0.9%)	2 (0.9%)
Patient physically incapable	6 (5.7%)	7 (6.5%)	13 (6.1%)
Patient excessively fatigued	1 (1.0%)	0 (0%)	1 (0.5%)
Patient refusal	1 (1.0%)	2 (1.9%)	3 (1.4%)
Unable to reach[b]	8 (7.6%)	5 (4.6%)	13 (6.1%)
Other	2 (1.9%)	1 (0.9%)	3 (1.4%)
**Digit Span**			
Patient Died[a]	0 (0%)	1 (0.9%)	1 (0.5%)
Patient Withdrew	1 (1.0%)	2 (1.9%)	3 (1.4%)
Suspected drug/alcohol influence	0 (0%)	0 (0%)	0 (0%)
Patient cognitively incapable	5 (4.8%)	8 (7.4%)	13 (6.1%)
Patient emotionally incapable	1 (1.0%)	1 (0.9%)	2 (0.9%)
Patient physically incapable	6 (5.7%)	9 (8.3%)	15 (7.0%)
Patient excessively fatigued	2 (1.9%)	3 (2.8%)	5 (2.3%)
Patient refusal	4 (3.8%)	4 (3.7%)	8 (3.8%)
Unable to reach[b]	9 (8.6%)	5 (4.6%)	14 (6.6%)
Other	3 (2.9%)	1 (0.9%)	4 (1.9%)
**WAIS-IV Similarities**			
Patient Died[a]	0 (0%)	1 (0.9%)	1 (0.5%)
Patient Withdrew	1 (1.0%)	2 (1.9%)	3 (1.4%)
Suspected drug/alcohol influence	0 (0%)	0 (0%)	0 (0%)
Patient cognitively incapable	5 (4.8%)	9 (8.3%)	14 (6.6%)
Patient emotionally incapable	1 (1.0%)	1 (0.9%)	2 (0.9%)
Patient physically incapable	6 (5.7%)	9 (8.3%)	15 (7.0%)

© 2023 Williams Roberson S et al. *JAMA Network Open.*

	Control (N=105)	Treatment (N=108)	Overall (N=213)
Patient excessively fatigued	1 (1.0%)	1 (0.9%)	2 (0.9%)
Patient refusal	2 (1.9%)	4 (3.7%)	6 (2.8%)
Unable to reach[b]	11 (10.5%)	8 (7.4%)	19 (8.9%)
Other	1 (1.0%)	1 (0.9%)	2 (0.9%)
**Hayling**			
Patient Died[a]	0 (0%)	1 (0.9%)	1 (0.5%)
Patient Withdrew	1 (1.0%)	2 (1.9%)	3 (1.4%)
Suspected drug/alcohol influence	0 (0%)	0 (0%)	0 (0%)
Patient cognitively incapable	5 (4.8%)	9 (8.3%)	14 (6.6%)
Patient emotionally incapable	1 (1.0%)	1 (0.9%)	2 (0.9%)
Patient physically incapable	6 (5.7%)	10 (9.3%)	16 (7.5%)
Patient excessively fatigued	2 (1.9%)	3 (2.8%)	5 (2.3%)
Patient refusal	4 (3.8%)	8 (7.4%)	12 (5.6%)
Unable to reach[b]	12 (11.4%)	9 (8.3%)	21 (9.9%)
Other	4 (3.8%)	2 (1.9%)	6 (2.8%)
**COWA**			
Patient Died[a]	0 (0%)	1 (0.9%)	1 (0.5%)
Patient Withdrew	1 (1.0%)	2 (1.9%)	3 (1.4%)
Suspected drug/alcohol influence	0 (0%)	0 (0%)	0 (0%)
Patient cognitively incapable	5 (4.8%)	9 (8.3%)	14 (6.6%)
Patient emotionally incapable	1 (1.0%)	2 (1.9%)	3 (1.4%)
Patient physically incapable	6 (5.7%)	10 (9.3%)	16 (7.5%)
Patient excessively fatigued	1 (1.0%)	1 (0.9%)	2 (0.9%)
Patient refusal	3 (2.9%)	5 (4.6%)	8 (3.8%)
Unable to reach[b]	11 (10.5%)	8 (7.4%)	19 (8.9%)
Other	2 (1.9%)	1 (0.9%)	3 (1.4%)
**Logical Memory I**			
Patient Died[a]	0 (0%)	1 (0.9%)	1 (0.5%)
Patient Withdrew	1 (1.0%)	2 (1.9%)	3 (1.4%)
Suspected drug/alcohol influence	0 (0%)	0 (0%)	0 (0%)

© 2023 Williams Roberson S et al. *JAMA Network Open.*

	Control (N=105)	Treatment (N=108)	Overall (N=213)
Patient cognitively incapable	5 (4.8%)	9 (8.3%)	14 (6.6%)
Patient emotionally incapable	1 (1.0%)	1 (0.9%)	2 (0.9%)
Patient physically incapable	6 (5.7%)	9 (8.3%)	15 (7.0%)
Patient excessively fatigued	2 (1.9%)	2 (1.9%)	4 (1.9%)
Patient refusal	3 (2.9%)	5 (4.6%)	8 (3.8%)
Unable to reach[b]	11 (10.5%)	6 (5.6%)	17 (8.0%)
Other	4 (3.8%)	1 (0.9%)	5 (2.3%)
**Logical Memory II**			
Patient Died[a]	0 (0%)	1 (0.9%)	1 (0.5%)
Patient Withdrew	1 (1.0%)	2 (1.9%)	3 (1.4%)
Suspected drug/alcohol influence	0 (0%)	0 (0%)	0 (0%)
Patient cognitively incapable	5 (4.8%)	9 (8.3%)	14 (6.6%)
Patient emotionally incapable	1 (1.0%)	1 (0.9%)	2 (0.9%)
Patient physically incapable	6 (5.7%)	10 (9.3%)	16 (7.5%)
Patient excessively fatigued	4 (3.8%)	2 (1.9%)	6 (2.8%)
Patient refusal	3 (2.9%)	6 (5.6%)	9 (4.2%)
Unable to reach[b]	12 (11.4%)	9 (8.3%)	21 (9.9%)
Other	9 (8.6%)	6 (5.6%)	15 (7.0%)

[a] Partial follow-up information (ADLs, FAQ and hospital utilization) were gathered from the surrogate for one participant who was hospitalized at the time of follow-up. This participant subsequently died prior to completing cognitive and psychological assessments, which were imputed based on previously gathered data.
[b] Participants designated as Unable to Reach at the individual assessment level were those for whom follow-up assessments had been initiated but not completed in one session, and a second session could not be arranged within the follow-up window.

© 2023 Williams Roberson S et al. *JAMA Network Open.*

eTable 3: Outcomes at 6 Months in Sepsis Survivors with and without Vitamin C, Thiamine & Hydrocortisone Therapy

**Cognitive Assessments, *median (IQR)***	**N[a]**	**Control, No. (%)**	**Treatment, No. (%)**
TICS	175	32 (29-34)	31 (25-34)
WAIS-IV Digit Span	161	9 (6-11)	8 (5-10)
WAIS-IV Similarities	163	8 (5-10)	7 (5-10)
Hayling Sentence Completion	147	4 (2-6)	4 (2-5)
COWA T-score	158	80 (31-46)	78 (27-46)
WMS-IV Logical Memory I (Immediate)	159	8 (5-11)	7 (4-9)
WMS-IV Logical Memory II (Delayed)	140	8 (5-10)	6 (4-8)
**Psychological Outcomes**	**N**	**Control**	**Treatment**
PTSD, *No. (%)*[b]	141	10 (9.5%)	18 (16.7%)
PTSD Symptom Burden, *median (IQR)*[c]	141	3 (2-8)	4 (0-8)
Depression, *No. (%)*[d]	142	27 (25.7%)	23 (21.3%)
Depression Symptom Burden, *median (IQR)*[e]	142	10 (7-16)	10 (6-16)
**Functional Outcomes, *median (IQR)***	**N**	**Control**	**Treatment**
ADLS	201	1 (0-4)	1 (0-5)
FAQ	199	4 (0-12)	4 (0-19)
Quality of Life (EQ-5D)	144	70 (50-85)	70 (50-85)
**Healthcare Utilization, *No. (%)***	**N**	**Control**	**Treatment**
Rehospitalization	209	37 (35.2%)	43 (39.8%)
Repeat ICU Stay	209	11 (10.5%)	14 (13.0%)
Received Mental Health Care	210	22 (21.0%)	13 (12.0%)
Medications for Depression or Anxiety	209	42 (40.0%)	36 (33.3%)

Where provided, percentages are of total participants analyzed in each treatment arm (105 for control group, 108 for treatment group).
Abbreviations: ADLS, Activities of Daily Living Scale; CI, Confidence Interval; COWA, Controlled Oral Word Association; EQ-5D, EuroQoL-5 Dimensions; FAQ, Functional Activities Questionnaire; ICU, Intensive Care Unit; IQR, Interquartile range; *n(%)*, number meeting criteria and percent of treatment arm; PTSD, Post-Traumatic Stress Disorder; TICS, Telephone Interview for Cognitive Status; WAIS, Wechsler Adult Intelligence Scale; WMS, Wechsler Memory Scale.

[a] N=Total number completing assessment

[b] Presence based on screening positive for PTSD using the PTSD-8 screening instrument (rating of 3 or more in 3 domains – see Supplementary Material).

[c] Symptom burden reported as sum of ratings of PTSD-related symptoms reported using PTSD-8 screening instrument (see Supplement 1)

[d] Presence based on using the Patient-Reported Outcome Measures (PROMIS)-6a Depression Short Form (T-score ≥ 60, corresponding with moderate depression)

[e] Symptom burden reported as sum of ratings of depression-related symptoms reported using PROMIS-6a Depression Short Form

© 2023 Williams Roberson S et al. *JAMA Network Open.*

eAppendix 3: Evaluation of the Proportional Odds Assumption and Probability Plots

We used a series of partial effects plots (demonstrating distributions of each model covariate as a function of levels for each outcome variable) to evaluate the proportional odds assumption. Based on our plots (data not shown), the proportional odds assumption holds reasonably well given the covariates in the model.

The following figure depicts the proportional odds probability P(Y>=y) as a function of outcome level y for each ordinal outcome. Orange lines represent probabilities for the control group and teal lines represent probabilities for the treatment group. Binary outcomes (PTSD and Depression) are excluded from this plot series as they are not ordinal in nature.

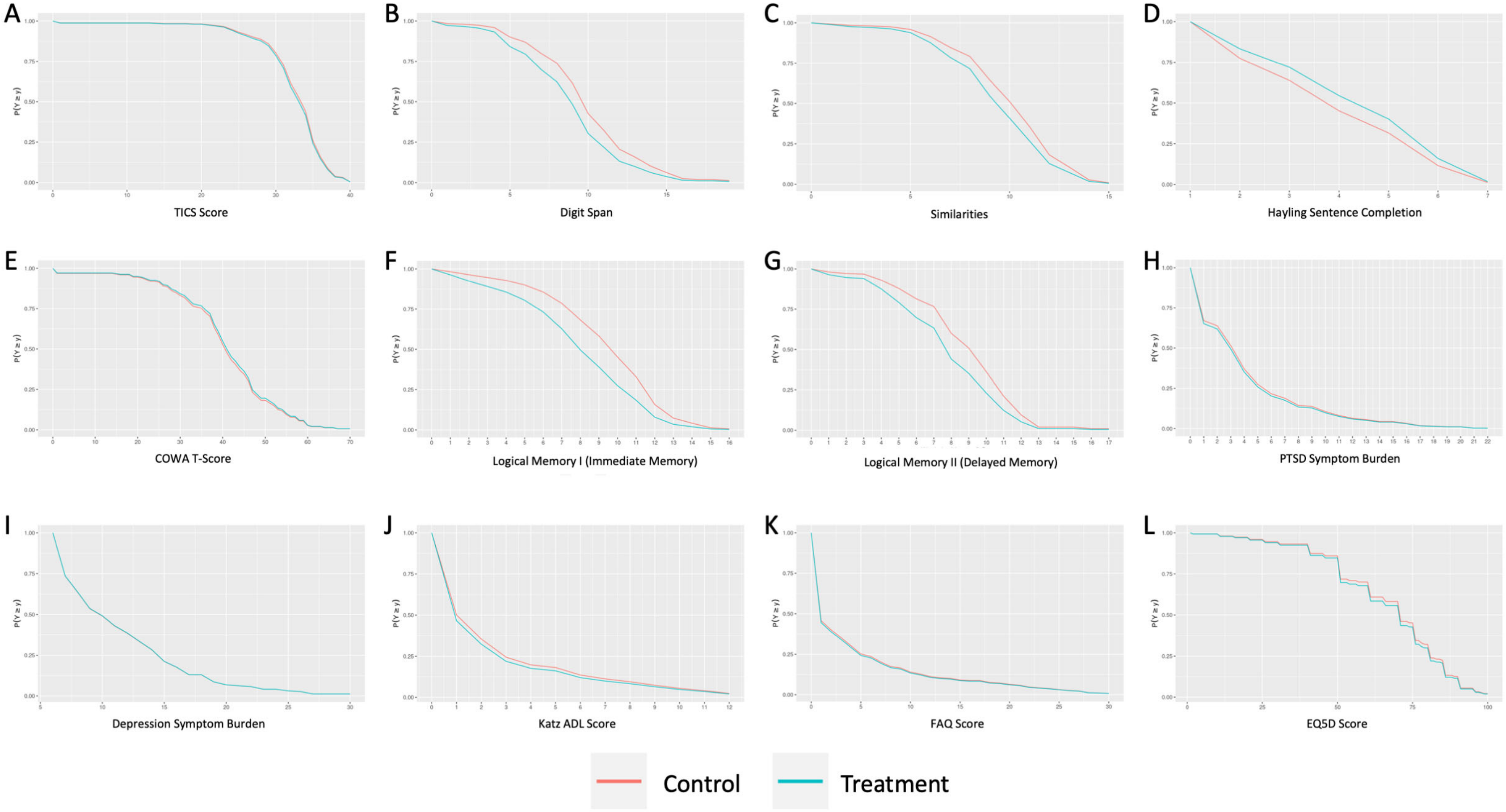

© 2023 Williams Roberson S et al. *JAMA Network Open.*

eAppendix 4: Adjusted Models (Complete Case Analysis)

All models in eTable 4 are based on a complete case approach (i.e. no imputation). Outcome variables are treated as ordinal N(25th-75th) or dichotomous N(%). To investigate the relationship between each outcome and randomization group an adjusted proportional odds or logistic model was fit. The point estimates from each model are summarized with adjusted Odds Ratios (OR), 95% confidence intervals, and p-value. The adjusted odds ratio (OR) is the odds of having an increased score or incurring the event of interest when the treatment group is compared to the control group. All models adjust for the following covariates: Treatment, Age, Sex, Race, Education, APACHE II, Presence of Diabetes/Neuro disease/Cardiovascular disease, days on ventilator, ICU length of stay.

© 2023 Williams Roberson S et al. *JAMA Network Open.*

eTable 4: Effect of Vitamin C, Thiamine & Hydrocortisone on 6-Month Outcomes after Sepsis

Outcome	N for analysis[a]	Control	Treatment	OR	95% C.I.	P-value
**Cognitive Performances**						
TICS Total Score	156	32 (29-34)	31 (25-34)	0.909	0.504 - 1.639	0.750
Digit Span Score	142	9 (7-11)	8 (5-10)	0.585	0.306 - 1.12	0.106
Similarities Score	145	8 (6-10)	8 (5-10)	0.66	0.359 - 1.214	0.181
Hayling Score	129	5 (2-6)	4 (2-5)	1.452	0.724 - 2.91	0.294
COWAT Score	140	8 (5-10)	7 (5-9)	0.999	0.527 - 1.896	0.998
Logical Memory1 Score	141	9 (6-11)	7 (5-9)	0.458	0.237 - 0.887	0.021
Logical Memory2 Score	126	8 (5-10)	6 (4-8)	0.525	0.267 - 1.033	0.062
**Psychological Status**						
PTSD-8 Symptom Burden	128	3 (1-9)	4 (0-8)	0.919	0.479 - 1.763	0.8
**PTSD	128	10 (15.15%)	14 (22.58%)	2.332	0.739 - 7.354	0.149
PROMIS Depression 6 Score	129	10 (7-15)	10 (6-15)	1.007	0.521 - 1.943	0.984
**Depression	129	24 (35.82%)	21 (33.87%)	1.277	0.515 - 3.166	0.598
**Functional Status**						
ADL Score	181	1 (0-4)	1 (0-4)	0.869	0.475 - 1.588	0.647
FAQ Score	179	4 (0-11)	4 (0-19)	0.952	0.541 - 1.675	0.863
EuroQoL 5D Score	131	70 (50-85)	74 (50-85)	0.902	0.473 - 1.721	0.755
**Healthcare Utilization**						
**Required mental health care	210	20 (21.98%)	12 (12.24%)	0.345	0.137 - 0.866	0.024
**Required medication for depression or anxiety	209	37 (41.11%)	33 (33.67%)	0.687	0.341 - 1.384	0.294

© 2023 Williams Roberson S et al. *JAMA Network Open.*

Outcome	N for analysis[a]	Control	Treatment	OR	95% C.I.	P-value
**Required rehospitalization	209	33 (36.67%)	37 (37.76%)	1.094	0.564 - 2.124	0.790
**Required repeat ICU stay	209	10 (30.30%)	11 (29.73%)	1.126	0.347 - 3.652	0.843

[a] N = Number of participants completing assessment. This may differ from number in primary analysis due to exclusion of participants who were cognitively unable to complete the assessment (in primary analysis these participants were assigned the lowest score). For dichotomous outcomes (marked with **), N indicates number meeting criteria for the outcome of interest (e.g. number of participants screening positive for PTSD). Positive PTSD screen was defined as a score of 3 or higher on 3 out of 4 symptom categories represented within the PTSD-8 questionnaire (see Hayley et al, . Depression was defined as a T-score of 60 or greater on the PROMIS-6 questionnaire.

© 2023 Williams Roberson S et al. *JAMA Network Open.*

eReferences

1. Marcantonio ER, Michaels M, Resnick NM. Diagnosing delirium by telephone. *J Gen Intern Med.* 1998;13(9):621-623.
2. Brandt J, Spencer M, Folstein MF. The Telephone Interview for Cognitive Status. *Neuropsychiatry, Neuropsychology and Behavioral Neurology.* 1988;1(2):111-117.
3. Weschsler D. *Wechsler Adult Intelligence Scale, 4th ed.* San Antonio, TX: Pearson; 2008.
4. Weschsler D. *Wechsler Memory Scale, 4th ed.* San Antonio, TX: Pearson; 2008.
5. Tombaugh TN, Kozak J, Rees L. Normative data stratified by age and education for two measures of verbal fluency: FAS and animal naming. *Arch Clin Neuropsychol.* 1999;14(2):167-177.
6. Burgess PW, Shallice T. *The Hayling and Brixton tests.* Bury St Edmunds: Thames Valley Test Company; 1997.
7. Hansen M, Andersen TE, Armour C, Elklit A, Palic S, Mackrill T. PTSD-8: A Short PTSD Inventory. *Clin Pract Epidemiol Ment Health.* 2010;6:101-108.
8. Mollica RF, Caspi-Yavin Y, Bollini P, Truong T, Tor S, Lavelle J. The Harvard Trauma Questionnaire. Validating a cross-cultural instrument for measuring torture, trauma, and posttraumatic stress disorder in Indochinese refugees. *J Nerv Ment Dis.* 1992;180(2):111-116.
9. Cella D, Yount S, Rothrock N, et al. The Patient-Reported Outcomes Measurement Information System (PROMIS): progress of an NIH Roadmap cooperative group during its first two years. *Med Care.* 2007;45(5 Suppl 1):S3-S11.
10. Choi SW, Schalet B, Cook KF, Cella D. Establishing a common metric for depressive symptoms: linking the BDI-II, CES-D, and PHQ-9 to PROMIS depression. *Psychol Assess.* 2014;26(2):513-527.
11. Katz S, Ford AB, Moskowitz RW, Jackson BA, Jaffe MW. Studies of Illness in the Aged. The Index of Adl: A Standardized Measure of Biological and Psychosocial Function. *JAMA : the journal of the American Medical Association.* 1963;185:914-919.
12. Pfeffer RI, Kurosaki TT, Harrah CH, Jr., Chance JM, Filos S. Measurement of functional activities in older adults in the community. *J Gerontol.* 1982;37(3):323-329.
13. Rabin R, de Charro F. EQ-5D: a measure of health status from the EuroQol Group. *Ann Med.* 2001;33(5):337-343.

© 2023 Williams Roberson S et al. *JAMA Network Open.*
