# Supplementary material for: Association of Vitamin C, Thiamine, and Hydrocortisone Infusion With Long-term Cognitive, Psychological, and Functional Outcomes in Sepsis Survivors: A Secondary Analysis of the Vitamin C, Thiamine, and Steroids in Sepsis Randomized Clinical Trial
Source: JAMA Netw Open. 2023 Feb 28;6(2):e230380. doi: 10.1001/jamanetworkopen.2023.0380 (PMC9975932; doi:10.1001/jamanetworkopen.2023.0380)
Supplement: Supplement 2. — Nonauthor Collaborators [file jamanetwopen-e230380-s002.pdf]

Supplemental Online Content: Nonauthor Collaborators
*First name, last name, and suffix (if applicable) are required and will appear in PubMed.

*Group Name(s): VICTAS Investigators							
***First Name and Middle Initial(s)**	***Last Name**	***Suffix (eg, Jr, III)**	Academic Degrees	Institution	Location (city, state/province, country)	Role or Contribution, eg, chair, principal investigator	Group (if more than 1 Group listed in the byline) and/or Subgroup (eg, Steering Committee)
Katherine	Nugent		MD	Emory University Hospital	Atlanta, Georgia, US	Site Principal Investigator	
Christine	Spainhour			Emory University Hospital	Atlanta, Georgia, US	Primary Study Coordinator	
Carmen	Polito		MD	Grady Memorial Hospital	Atlanta, Georgia, US	Site Principal Investigator	
Brooks	Moore		MD	Grady Memorial Hospital	Atlanta, Georgia, US	Site Co-Investigator	
Lovie	Negrin			Grady Memorial Hospital	Atlanta, Georgia, US	Primary Study Coordinator	
Akram	Khan		MD	Oregon Health Sciences University	Portland, Oregon, US	Site Principal Investigator	
Bory	Kea		MD	Oregon Health Sciences University	Portland, Oregon, US	Site Co-Investigator	
Olivia	Krol			Oregon Health Sciences University	Portland, Oregon, US	Primary Study Coordinator	
Ebaad	Haq			Oregon Health Sciences University	Portland, Oregon, US	Other Research Team	
Vincent	Pinkert			Oregon Health Sciences University	Portland, Oregon, US	Other Research Team	
Kelly	Nguyen			Oregon Health Sciences University	Portland, Oregon, US	Other Research Team	
Samuel M	Brown		MD	Intermountain Medical Center	Murray, Utah, US	Site Principal Investigator	
Joseph	Bledsoe		MD	Intermountain Medical Center	Murray, Utah, US	Site Co-Investigator	
Ithan	Peltan		MD	Intermountain Medical Center	Murray, Utah, US	Site Co-Investigator	
Darrin	Applegate			Intermountain Medical Center	Murray, Utah, US	Primary Study Coordinator	

Supplemental Online Content: Nonauthor Collaborators
*First name, last name, and suffix (if applicable) are required and will appear in PubMed.

*First Name and Middle Initial(s)	*Last Name	*Suffix (eg, Jr, III)	Academic Degrees	Institution	Location (city, state/province, country)	Role or Contribution, eg, chair, principal investigator	Group (if more than 1 Group listed in the byline) and/or Subgroup (eg, Steering Committee)
Brent	Armbuster			Intermountain Medical Center	Murray, Utah, US	Other Research Team	
Quinn	Montgomery			Intermountain Medical Center	Murray, Utah, US	Other Research Team	
Katie	Brown			Intermountain Medical Center	Murray, Utah, US	Other Research Team	
Austin	Daw			Intermountain Medical Center	Murray, Utah, US	Other Research Team	
Michelle	Gong		MD	Montefiore Medical Center	Bronx, New York, US	Site Principal Investigator	Moses, Weiler
Michael	Aboodi		MD	Montefiore Medical Center	Bronx, New York, US	Site Co-Investigator	Moses, Weiler
Jen-Ting (Tina)	Chen		MD	Montefiore Medical Center	Bronx, New York, US	Site Co-Investigator	Moses, Weiler
Aluko	Hope		MD	Montefiore Medical Center	Bronx, New York, US	Site Co-Investigator	Moses, Weiler
Swarna	Gummadi			Montefiore Medical Center	Bronx, New York, US	Primary Study Coordinator	Moses
Brenda	Lopez			Montefiore Medical Center	Bronx, New York, US	Primary Study Coordinator	Weiler
Brenda	Lopez			Montefiore Medical Center	Bronx, New York, US	Other Research Team	Moses
Jeremiah	Hinson		MD	Johns Hopkins Hospital	Baltimore, Maryland, US	Site Principal Investigator	
David	Hager		MD	Johns Hopkins Hospital	Baltimore, Maryland, US	Site Co-Investigator	
Erin	Ricketts			Johns Hopkins Hospital	Baltimore, Maryland, US	Primary Study Coordinator	
David	Hager		MD	Johns Hopkins Bayview	Baltimore, Maryland, US	Site Principal Investigator	

Supplemental Online Content: Nonauthor Collaborators
*First name, last name, and suffix (if applicable) are required and will appear in PubMed.

*First Name and Middle Initial(s)	*Last Name	*Suffix (eg, Jr, III)	Academic Degrees	Institution	Location (city, state/province, country)	Role or Contribution, eg, chair, principal investigator	Group (if more than 1 Group listed in the byline) and/or Subgroup (eg, Steering Committee)
Johnathan	Clare		MD	Johns Hopkins Bayview	Baltimore, Maryland, US	Site Co-Investigator	
Breana	McBryde			Johns Hopkins Bayview	Baltimore, Maryland, US	Primary Study Coordinator	
Casey M.	Clements		MD	Mayo Clinic	Rochester, Minnesota, US	Site Principal Investigator	
Ognjen	Gajic		MD	Mayo Clinic	Rochester, Minnesota, US	Site Co-Investigator	
Rahul	Kashyap		MD	Mayo Clinic	Rochester, Minnesota, US	Site Co-Investigator	
Derek	Vanmeter			Mayo Clinic	Rochester, Minnesota, US	Primary Study Coordinator	
Laurence	Busse		MD	Emory Saint Joseph's Hospital	Atlanta, Georgia, US	Site Principal Investigator	
Mary	McBride			Emory Saint Joseph's Hospital	Atlanta, Georgia, US	Primary Study Coordinator	
Adit	Ginde		MD	University of Colorado School of Med	Boulder, Colorado, US	Site Principal Investigator	
Marc	Moss		MD	University of Colorado School of Med	Boulder, Colorado, US	Site Co-Investigator	
Lani	Finck			University of Colorado School of Med	Boulder, Colorado, US	Primary Study Coordinator	
Michelle	Howell			University of Colorado School of Med	Boulder, Colorado, US	Other Research Team	
Jeffrey	McKeehan			University of Colorado School of Med	Boulder, Colorado, US	Other Research Team	
Carrie	Higgins			University of Colorado School of Med	Boulder, Colorado, US	Other Research Team	
Aaron	Barksdale		MD	University of Nebraska Medical Cent	Omaha, Nebraska, US	Site Principal Investigator	

Supplemental Online Content: Nonauthor Collaborators
*First name, last name, and suffix (if applicable) are required and will appear in PubMed.

*First Name and Middle Initial(s)	*Last Name	*Suffix (eg, Jr, III)	Academic Degrees	Institution	Location (city, state/province, country)	Role or Contribution, eg, chair, principal investigator	Group (if more than 1 Group listed in the byline) and/or Subgroup (eg, Steering Committee)
Dan	Kalin		MD	University of Nebraska Medical Cente	Omaha, Nebraska, US	Site Co-Investigator	
Derek	Kruse		MD	University of Nebraska Medical Cente	Omaha, Nebraska, US	Site Co-Investigator	
Katlyn	Hilz			University of Nebraska Medical Cente	Omaha, Nebraska, US	Primary Study Coordinator	
Nida	Qadir		MD	David Geffen School of Medicine at U	Los Angeles, California, US	Site Principal Investigator	
Steven Y.	Chang		MD	David Geffen School of Medicine at U	Los Angeles, California, US	Site Co-Investigator	
Rebecca	Beutler			David Geffen School of Medicine at U	Los Angeles, California, US	Primary Study Coordinator	
Andrea	Tam			David Geffen School of Medicine at U	Los Angeles, California, US	Other Research Team	
Estelle S.	Harris		MD	University of Utah	Salt Lake City, Utah, US	Site Principal Investigator	
Scott T.	Youngquist		MD	University of Utah	Salt Lake City, Utah, US	Site Co-Investigator	
Elizabeth A.	Middleton		MD	University of Utah	Salt Lake City, Utah, US	Site Co-Investigator	
Ervin	Davidov			University of Utah	Salt Lake City, Utah, US	Primary Study Coordinator	
Amber	Plante			University of Utah	Salt Lake City, Utah, US	Other Research Team	
Justin	Belsky		MD	Yale New Haven Hospital	New Haven, Conneticut, US	Site Principal Investigator	
Jonathan	Siner		MD	Yale New Haven Hospital	New Haven, Conneticut, US	Site Co-Investigator	
Charles	Wira		MD	Yale New Haven Hospital	New Haven, Conneticut, US	Site Co-Investigator	

Supplemental Online Content: Nonauthor Collaborators
*First name, last name, and suffix (if applicable) are required and will appear in PubMed.

***First Name and Middle Initial(s)**	***Last Name**	***Suffix (eg, Jr, III)**	Academic Degrees	Institution	Location (city, state/province, country)	Role or Contribution, eg, chair, principal investigator	Group (if more than 1 Group listed in the byline) and/or Subgroup (eg, Steering Committee)
Carolyn	Brokowski			Yale New Haven Hospital	New Haven, Conneticut, US	Primary Study Coordinator	
Jay	Steingrub		MD	Baystate Health	Springfield, Massachusetts, US	Site Principal Investigator	
Howard	Smithline		MD	Baystate Health	Springfield, Massachusetts, US	Site Co-Investigator	
Sherell	Thorton-Thompson			Baystate Health	Springfield, Massachusetts, US	Primary Study Coordinator	
Alpha	Fowler		MD	Virginia Commonwealth University	Richmond, Virginia, US	Site Principal Investigator	
Stephen	Miller		DO	Virginia Commonwealth University	Richmond, Virginia, US	Site Co-Investigator	
Kyle	Narron			Virginia Commonwealth University	Richmond, Virginia, US	Primary Study Coordinator	
Michael A.	Pusckarich		MD	Hennepin County Medical Center	Minneapolis, Massachusetts, US	Site Principal Investigator	
Matthew E.	Prekker		MD	Hennepin County Medical Center	Minneapolis, Massachusetts, US	Site Co-Investigator	
Audrey	Hendrickson			Hennepin County Medical Center	Minneapolis, Massachusetts, US	Primary Study Coordinator	
James	Quinn		MD	Stanford University	Stanford, California, US	Site Principal Investigator	
Jennifer	Wilson		MD	Stanford University	Stanford, California, US	Site Co-Investigator	
Joseph	Levitt		MD	Stanford University	Stanford, California, US	Site Co-Investigator	
Rosen	Mann			Stanford University	Stanford, California, US	Primary Study Coordinator	
Anita	Visweswaran			Stanford University	Stanford, California, US	Other Research Team	

Supplemental Online Content: Nonauthor Collaborators
*First name, last name, and suffix (if applicable) are required and will appear in PubMed.

*First Name and Middle Initial(s)	*Last Name	*Suffix (eg, Jr, III)	Academic Degrees	Institution	Location (city, state/province, country)	Role or Contribution, eg, chair, principal investigator	Group (if more than 1 Group listed in the byline) and/or Subgroup (eg, Steering Committee)
Nina	Gentile		MD	Temple University	Philadelphia, Pennsylvania, US	Site Principal Investigator	
Nathaniel	Marchetti		DO	Temple University	Philadelphia, Pennsylvania, US	Site Co-Investigator	
Hannah	Reimer			Temple University	Philadelphia, Pennsylvania, US	Primary Study Coordinator	
Faheem	Guirgis		MD	University of Florida Jacksonville	Jacksonville, Florida, US	Site Principal Investigator	
Lisa	Jones		MD	University of Florida Jacksonville	Jacksonville, Florida, US	Site Co-Investigator	
Lauren	Black		MD	University of Florida Jacksonville	Jacksonville, Florida, US	Site Co-Investigator	
Morgan	Henson			University of Florida Jacksonville	Jacksonville, Florida, US	Primary Study Coordinator	
Nuala J.	Meyer		MD	University of Pennsylvania Health Sys	Philadelphia, Pennsylvania, US	Site Principal Investigator	
John C.	Greenwood		MD	University of Pennsylvania Health Sys	Philadelphia, Pennsylvania, US	Site Co-Investigator	
Caroline	Ittner			University of Pennsylvania Health Sys	Philadelphia, Pennsylvania, US	Primary Study Coordinator	
Emanuel	Rivers		MD	Henry Ford Health System	Detroit, Michigan, US	Site Principal Investigator	
Namita	Jayaprakash		MD	Henry Ford Health System	Detroit, Michigan, US	Site Co-Investigator	
Jayna	Gardner-Gray		MD	Henry Ford Health System	Detroit, Michigan, US	Site Co-Investigator	
Gina	Hurst		MD	Henry Ford Health System	Detroit, Michigan, US	Site Co-Investigator	
Jacqueline	Pflaum		MD	Henry Ford Health System	Detroit, Michigan, US	Site Co-Investigator	

Supplemental Online Content: Nonauthor Collaborators
*First name, last name, and suffix (if applicable) are required and will appear in PubMed.

***First Name and Middle Initial(s)**	***Last Name**	***Suffix (eg, Jr, III)**	Academic Degrees	Institution	Location (city, state/province, country)	Role or Contribution, eg, chair, principal investigator	Group (if more than 1 Group listed in the byline) and/or Subgroup (eg, Steering Committee)
Anja Kathrin	Jaehne			Henry Ford Health System	Detroit, Michigan, US	Primary Study Coordinator	
Jasreen	Gill			Henry Ford Health System	Detroit, Michigan, US	Other Research Team	
Aaron	Cook			Henry Ford Health System	Detroit, Michigan, US	Other Research Team	
David R	Janz		MD	Louisana State University	Baton Rouge, Louisiana, US	Site Principal Investigator	
Derek	Vonderhaar		MD	Louisana State University	Baton Rouge, Louisiana, US	Site Co-Investigator	
Connie	Romaine			Louisana State University	Baton Rouge, Louisiana, US	Primary Study Coordinator	
R. Gentry	Wilkerson		MD	University of Maryland School of Me	Baltimore, Maryland, US	Site Principal Investigator	
Michael T.	McCurdy		MD	University of Maryland School of Me	Baltimore, Maryland, US	Site Co-Investigator	
Dana	Beach			University of Maryland School of Me	Baltimore, Maryland, US	Primary Study Coordinator	
Kyra	Lasko			University of Maryland School of Me	Baltimore, Maryland, US	Other Research Team	
Richard	Gill		MD	Bon Secours	Greenville, South Carolina, US	Site Principal Investigator	
Katherine	Price		MD	Bon Secours	Greenville, South Carolina, US	Site Co-Investigator	
Lisa	Dickson			Bon Secours	Greenville, South Carolina, US	Primary Study Coordinator	
Abhijit	Duggal		MD	Cleveland Clinic	Cleveland, Ohio, US	Site Principal Investigator	
Sharon E.	Mace		MD	Cleveland Clinic	Cleveland, Ohio, US	Site Co-Investigator	

Supplemental Online Content: Nonauthor Collaborators
*First name, last name, and suffix (if applicable) are required and will appear in PubMed.

***First Name and Middle Initial(s)**	***Last Name**	***Suffix (eg, Jr, III)**	Academic Degrees	Institution	Location (city, state/province, country)	Role or Contribution, eg, chair, principal investigator	Group (if more than 1 Group listed in the byline) and/or Subgroup (eg, Steering Committee)
R. Duncan	Hite		MD	Cleveland Clinic	Cleveland, Ohio, US	Site Co-Investigator	
Andrei	Hastings			Cleveland Clinic	Cleveland, Ohio, US	Primary Study Coordinator	
Jason	Haukoos		MD	Denver Health	Denver, Colorado, US	Site Principal Investigator	
Ivor	Dougla		MD	Denver Health	Denver, Colorado, US	Site Co-Investigator	
Stacy	Trent		MD	Denver Health	Denver, Colorado, US	Site Co-Investigator	
Carolynn	Lyle			Denver Health	Denver, Colorado, US	Primary Study Coordinator	
Alicia	Cupelo			Denver Health	Denver, Colorado, US	Other Research Team	
Stephanie	Gravitz			Denver Health	Denver, Colorado, US	Other Research Team	
Terra	Hiller			Denver Health	Denver, Colorado, US	Other Research Team	
Judy	Oakes			Denver Health	Denver, Colorado, US	Other Research Team	
Frank	LoVecchio		DO	Maricopa Integrated Health System	Phoenix, Arizona, US	Site Principal Investigator	
Pedro	Quiroga		MD	Maricopa Integrated Health System	Phoenix, Arizona, US	Site Co-Investigator	
Shiloh	Danley			Maricopa Integrated Health System	Phoenix, Arizona, US	Site Co-Investigator	
Mary	Mulrow			Maricopa Integrated Health System	Phoenix, Arizona, US	Primary Study Coordinator	
Amanda	Encinas			Maricopa Integrated Health System	Phoenix, Arizona, US	Other Research Team	

Supplemental Online Content: Nonauthor Collaborators
*First name, last name, and suffix (if applicable) are required and will appear in PubMed.

***First Name and Middle Initial(s)**	***Last Name**	***Suffix (eg, Jr, III)**	Academic Degrees	Institution	Location (city, state/province, country)	Role or Contribution, eg, chair, principal investigator	Group (if more than 1 Group listed in the byline) and/or Subgroup (eg, Steering Committee)
Andrew	Goodwin		MD	Medical University of South Carolina	Charleston, South Carolina, US	Site Principal Investigator	
Gregory	Hall		MD	Medical University of South Carolina	Charleston, South Carolina, US	Site Co-Investigator	
Abigail	Grady			Medical University of South Carolina	Charleston, South Carolina, US	Primary Study Coordinator	
Matthew	Exline		MD	Ohio State University Wexner Medica	Columbus, Ohio, US	Site Principal Investigator	
Thomas	Terndrup		MD	Ohio State University Wexner Medica	Columbus, Ohio, US	Site Co-Investigator	
Sonal	Pannu		MD	Ohio State University Wexner Medica	Columbus, Ohio, US	Site Co-Investigator	
Emily	Robart			Ohio State University Wexner Medica	Columbus, Ohio, US	Primary Study Coordinator	
Sarah	Karow			Ohio State University Wexner Medica	Columbus, Ohio, US	Other Research Team	
D. Clark	Files		MD	Wake Forest University	Winston-Salem, North Carolina, US	Site Principal Investigator	
Lane	Smith		MD	Wake Forest University	Winston-Salem, North Carolina, US	Site Co-Investigator	
Kevin	Gibbs		MD	Wake Forest University	Winston-Salem, North Carolina, US	Site Co-Investigator	
Lori	Flores			Wake Forest University	Winston-Salem, North Carolina, US	Primary Study Coordinator	
Stephen M.	Pastores		MD	Memorial Sloan Kettering Cancer Cer	New York, New York, US	Site Principal Investigator	
David J.	Shaz		MD	Memorial Sloan Kettering Cancer Cer	New York, New York, US	Site Co-Investigator	
Natalie	Kostelecky			Memorial Sloan Kettering Cancer Cer	New York, New York, US	Primary Study Coordinator	

Supplemental Online Content: Nonauthor Collaborators
*First name, last name, and suffix (if applicable) are required and will appear in PubMed.

***First Name and Middle Initial(s)**	***Last Name**	***Suffix (eg, Jr, III)**	Academic Degrees	Institution	Location (city, state/province, country)	Role or Contribution, eg, chair, principal investigator	Group (if more than 1 Group listed in the byline) and/or Subgroup (eg, Steering Committee)
Chad	Chase		MD	Piedmont Healthcare	Atlanta, Georgia, US	Site Principal Investigator	
Elizabeth (Liz)	Wilkins			Piedmont Healthcare	Atlanta, Georgia, US	Primary Study Coordinator	
David	Gaieski		MD	Thomas Jefferson University	Philadelphia, Pennsylvania, US	Site Co-Investigator	
Michael	Baram		MD	Thomas Jefferson University	Philadelphia, Pennsylvania, US	Site Co-Investigator	
Daniel	Schwegler			Thomas Jefferson University	Philadelphia, Pennsylvania, US	Primary Study Coordinator	
Nicole	Renzi			Thomas Jefferson University	Philadelphia, Pennsylvania, US	Other Research Team	
Jarrod M.	Mosier		MD	University of Arizona	Tucson, Arizona, US	Site Principal Investigator	
Cameron	Hypes		MD	University of Arizona	Tucson, Arizona, US	Site Co-Investigator	
Elizabeth	Salvagio Campbell			University of Arizona	Tucson, Arizona, US	Primary Study Coordinator	
Michael	Hooper		MD	Senatara Healthcare	Norfolk, Virginia, US	Site Principal Investigator	
Joshua	Sill		MD	Senatara Healthcare	Norfolk, Virginia, US	Site Co-Investigator	
Kate	Mitchell			Senatara Healthcare	Norfolk, Virginia, US	Primary Study Coordinator	
Kristin	Hudock		MD	University of Cinnicinati Physicians Co	Cinnicinati, Ohio, US	Site Principal Investigator	
Michael	Lyons		MD	University of Cinnicinati Physicians Co	Cinnicinati, Ohio, US	Site Co-Investigator	
Kari	Gordner		MD	University of Cinnicinati Physicians Co	Cinnicinati, Ohio, US	Site Principal Investigator	

Supplemental Online Content: Nonauthor Collaborators

*First name, last name, and suffix (if applicable) are required and will appear in PubMed.

***First Name and Middle Initial(s)**	***Last Name**	***Suffix (eg, Jr, III)**	Academic Degrees	Institution	Location (city, state/province, country)	Role or Contribution, eg, chair, principal investigator	Group (if more than 1 Group listed in the byline) and/or Subgroup (eg, Steering Committee)
Yousef	Ahmad		DO	University of Cinnicinati Physicians Co	Cinnicinati, Ohio, US	Site Co-Investigator	
Autumn	Studer			University of Cinnicinati Physicians Co	Cinnicinati, Ohio, US	Primary Study Coordinator	
Jacqueline	Davis			University of Cinnicinati Physicians Co	Cinnicinati, Ohio, US	Other Research Team	
Matthew	Barrett		DO	Christiana Care	Newark, Delaware, US	Site Principal Investigator	
Jason	Nomura		MD	Christiana Care	Newark, Delaware, US	Site Co-Investigator	
Jennifer	Knox			Christiana Care	Newark, Delaware, US	Primary Study Coordinator	
Pauline	Park		MD	University of Michigan	Ann Arbor, Michigan, US	Site Principal Investigator	
Ivan	Co		MD	University of Michigan	Ann Arbor, Michigan, US	Site Co-Investigator	
Jakob	McSparron		MD	University of Michigan	Ann Arbor, Michigan, US	Site Principal Investigator	
Robert	Hyzy		MD	University of Michigan	Ann Arbor, Michigan, US	Site Co-Investigator	
Kelli	McDonough			University of Michigan	Ann Arbor, Michigan, US	Primary Study Coordinator	
Sinan	Hanna			University of Michigan	Ann Arbor, Michigan, US	Other Research Team	
Wesley H.	Self		MD	Vanderbilt University	Nashville, Tennessee, US	Site Principal Investigator	
Matthew W.	Semler		MD	Vanderbilt University	Nashville, Tennessee, US	Site Co-Investigator	
Margaret	Hays			Vanderbilt University	Nashville, Tennessee, US	Primary Study Coordinator	

Supplemental Online Content: Nonauthor Collaborators
*First name, last name, and suffix (if applicable) are required and will appear in PubMed.

***First Name and Middle Initial(s)**	***Last Name**	***Suffix (eg, Jr, III)**	Academic Degrees	Institution	Location (city, state/province, country)	Role or Contribution, eg, chair, principal investigator	Group (if more than 1 Group listed in the byline) and/or Subgroup (eg, Steering Committee)
Racquel	Bartz		MD	Duke University	Durham, North Carolina, US	Site Principal Investigator	
Alexander	Limkakeng		MD	Duke University	Durham, North Carolina, US	Site Co-Investigator	
Katherine	Sweeney			Duke University	Durham, North Carolina, US	Primary Study Coordinator	
Rachael	Woodburn			Duke University	Durham, North Carolina, US	Other Research Team	
Munish	Goyal		MD	Medstar Washington Hospital Center	Washington, DC, US	Site Principal Investigator	
Akram	Zaaqoq		MD	Medstar Washington Hospital Center	Washington, DC, US	Site Co-Investigator	
Theresa	Moriarty			Medstar Washington Hospital Center	Washington, DC, US	Primary Study Coordinator	
John	Oropello		MD	Mount Sinai Hospital	New York City, New York	Site Principal Investigator	
Ziya	Zhang			Mount Sinai Hospital	New York City, New York	Primary Study Coordinator	
