# Supplementary material for: Association of Vitamin C, Thiamine, and Hydrocortisone Infusion With Long-term Cognitive, Psychological, and Functional Outcomes in Sepsis Survivors: A Secondary Analysis of the Vitamin C, Thiamine, and Steroids in Sepsis Randomized Clinical Trial
Source: JAMA Netw Open. 2023 Feb 28;6(2):e230380. doi: 10.1001/jamanetworkopen.2023.0380 (PMC9975932; doi:10.1001/jamanetworkopen.2023.0380)
Supplement: Supplement 3. — Data Sharing Statement [file jamanetwopen-e230380-s003.pdf]

Data Sharing Statement

Williams Roberson. Association of Vitamin C, Thiamine, and Hydrocortisone Infusion With Long-term Cognitive, Psychological, and Functional Outcomes in Sepsis Survivors. *JAMA Netw Open*. Published February 28, 2023. doi:10.1001/jamanetworkopen.2023.0380

Data
Data available: No
